# Vaccination with structurally adapted fungal protein fibrils induces immunity to Parkinson’s disease

**DOI:** 10.1093/brain/awae061

**Published:** 2024-03-01

**Authors:** Verena Pesch, José Miguel Flores-Fernandez, Sara Reithofer, Liang Ma, Pelin Özdüzenciler, Yannick Busch, Aishwarya Sriraman, YongLiang Wang, Sara Amidian, Chiara V M Kroepel, Laura Müller, Yi Lien, Olivia Rudtke, Benedikt Frieg, Gunnar F Schröder, Holger Wille, Gültekin Tamgüney

**Affiliations:** Institut für Biologische Informationsprozesse, Strukturbiochemie (IBI-7), Forschungszentrum Jülich, 52425 Jülich, Germany; Department of Biochemistry and Centre for Prions and Protein Folding Diseases, University of Alberta, Edmonton, AB T6G 2M8, Canada; Institut für Biologische Informationsprozesse, Strukturbiochemie (IBI-7), Forschungszentrum Jülich, 52425 Jülich, Germany; Institut für Biologische Informationsprozesse, Strukturbiochemie (IBI-7), Forschungszentrum Jülich, 52425 Jülich, Germany; Institut für Biologische Informationsprozesse, Strukturbiochemie (IBI-7), Forschungszentrum Jülich, 52425 Jülich, Germany; Institut für Biologische Informationsprozesse, Strukturbiochemie (IBI-7), Forschungszentrum Jülich, 52425 Jülich, Germany; Department of Biochemistry and Centre for Prions and Protein Folding Diseases, University of Alberta, Edmonton, AB T6G 2M8, Canada; Department of Biochemistry and Centre for Prions and Protein Folding Diseases, University of Alberta, Edmonton, AB T6G 2M8, Canada; Department of Cell Biology and Anatomy, University of Calgary, Calgary, AB T2N 1N4, Canada; Department of Biochemistry and Centre for Prions and Protein Folding Diseases, University of Alberta, Edmonton, AB T6G 2M8, Canada; Institut für Biologische Informationsprozesse, Strukturbiochemie (IBI-7), Forschungszentrum Jülich, 52425 Jülich, Germany; Institut für Biologische Informationsprozesse, Strukturbiochemie (IBI-7), Forschungszentrum Jülich, 52425 Jülich, Germany; Institut für Biologische Informationsprozesse, Strukturbiochemie (IBI-7), Forschungszentrum Jülich, 52425 Jülich, Germany; Institut für Biologische Informationsprozesse, Strukturbiochemie (IBI-7), Forschungszentrum Jülich, 52425 Jülich, Germany; Institut für Biologische Informationsprozesse, Strukturbiochemie (IBI-7), Forschungszentrum Jülich, 52425 Jülich, Germany; Institut für Biologische Informationsprozesse, Strukturbiochemie (IBI-7), Forschungszentrum Jülich, 52425 Jülich, Germany; Physics Department, Heinrich-Heine-Universität Düsseldorf, 40225 Düsseldorf, Germany; Department of Biochemistry and Centre for Prions and Protein Folding Diseases, University of Alberta, Edmonton, AB T6G 2M8, Canada; Neuroscience and Mental Health Institute, University of Alberta, Edmonton, AB T6G 2M8, Canada; Institut für Biologische Informationsprozesse, Strukturbiochemie (IBI-7), Forschungszentrum Jülich, 52425 Jülich, Germany; Institut für Physikalische Biologie, Heinrich-Heine-Universität Düsseldorf, 40225 Düsseldorf, Germany

**Keywords:** alpha-synuclein, amyloid, conformation, immunization, fibril, vaccine

## Abstract

The pathological misfolding and aggregation of soluble α-synuclein into toxic oligomers and insoluble amyloid fibrils causes Parkinson’s disease, a progressive age-related neurodegenerative disease for which there is no cure.

HET-s is a soluble fungal protein that can form assembled amyloid fibrils in its prion state. We engineered HET-s(218–298) to form four different fibrillar vaccine candidates, each displaying a specific conformational epitope present on the surface of α-synuclein fibrils. Vaccination with these four vaccine candidates prolonged the survival of immunized TgM83^+/−^ mice challenged with α-synuclein fibrils by 8% when injected into the brain to model brain-first Parkinson’s disease or by 21% and 22% when injected into the peritoneum or gut wall, respectively, to model body-first Parkinson’s disease. Antibodies from fully immunized mice recognized α-synuclein fibrils and brain homogenates from patients with Parkinson’s disease, dementia with Lewy bodies and multiple system atrophy.

Conformation-specific vaccines that mimic epitopes present only on the surface of pathological fibrils but not on soluble monomers, hold great promise for protection against Parkinson’s disease, related synucleinopathies and other amyloidogenic protein misfolding disorders.

## Teaser

Misfolded α-synuclein forms toxic aggregates in Parkinson's disease. By modifying fungal protein fibrils to include surface regions of α-synuclein fibrils, Pesch *et al.* develop four Parkinson's disease vaccine candidates. Immunization of mouse models of Parkinson’s disease extended survival and improved motor function.

**See Kinoshita *et al*. (https://doi.org/10.1093/brain/awae115) for a scientific commentary on this article.**

## Introduction

α-Synuclein (α-syn) is a cytosolic protein that under physiological conditions is soluble. Pathologic aggregation of α-syn into toxic oligomers and insoluble amyloid fibrils causes Parkinson’s disease (PD), Lewy body dementia (LBD), multiple system atrophy (MSA) and other age-related progressive neurodegenerative diseases for which there is no cure.^1-3^ These diseases are characterized by deposits of pathological α-syn aggregates known as Lewy bodies in PD and LBD, and as glial cytoplasmic inclusions in MSA.

Pathological aggregates of α-syn have prion-like properties because they can spread from diseased to unaffected neurons and there seed *de novo* aggregation of α-syn by recruiting monomeric α-syn into growing fibrils. Large fibrils fracture and give rise to smaller seeds that feed this vicious cycle. Transmission of pathological α-syn aggregates between neurons has been found to be transsynaptic and may involve multiple mechanisms.^4^ Studies show that α-syn pathology can spread within the nervous system.^5,6^ The rostro-caudal spread of α-syn pathology within the CNS in PD is described by the Braak staging system.^6^ Additional evidence shows that α-syn pathology can spread from the enteric nervous system (ENS), which innervates the gastrointestinal tract, to the CNS, along peripheral nerves or in the blood.^7-11^

Because α-syn is a self-antigen, the immune system does not mount a sufficiently protective response to pathological α-syn aggregates. Advances in solid-state nuclear magnetic resonance (NMR) spectroscopy and electron cryo-microscopy (cryo-EM) have helped to solve the structures of synthetic^12,13^ and *ex vivo* α-syn fibrils isolated from patients’ brains.^14,15^ Here, we designed a quadrivalent vaccine to induce an immune response against pathological α-syn fibrils by molecular grafting of conformational epitopes present on α-syn fibrils onto the carrier molecule HET-s(218–289), the prion domain of HET-s found in the filamentous fungus *Podospora anserina.*^16^ We show in mouse models of PD that vaccination with this quadrivalent vaccine induces antibodies that recognize α-syn fibrils and protect against PD-like symptoms, resulting in improved motor performance and prolonged survival.

## Materials and methods

Full details of the methods are provided in the Supplementary material.

## Results

### HET-s can be modified to engineer fibrillar vaccine candidates that mimic pathological α-syn fibrils

**Figure 1:**
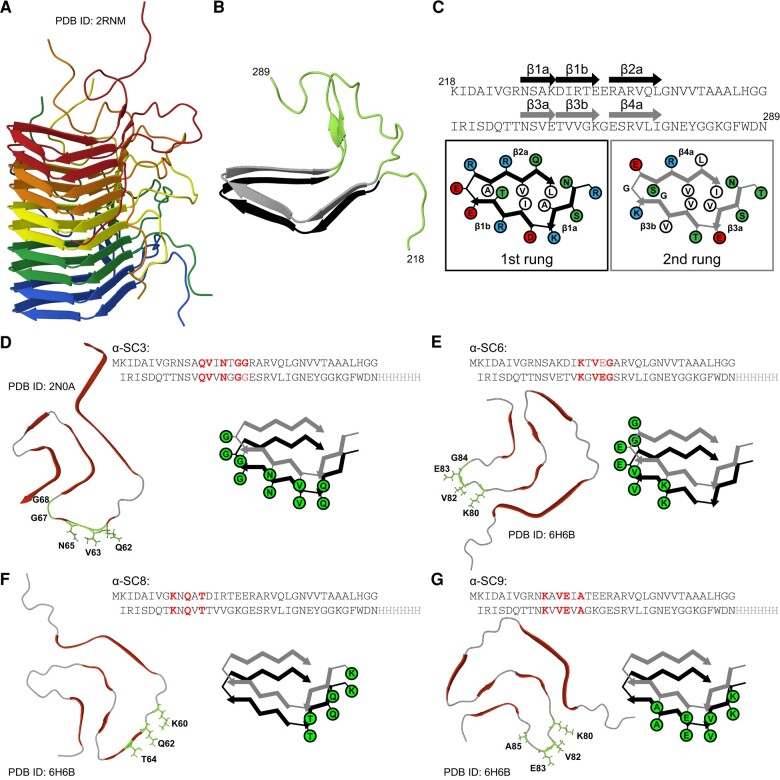
**HET-s-based vaccine candidates display conformational epitopes present on α-synuclein (α-syn) fibrils.** (**A**) HET-s(218–289) forms fibrils *in vitro*, shown here as a heptamer [protein data bank (PDB) ID: 2RNM].^18^ (**B**) In fibrillar HET-s(218–289) eight β-strands form two windings of a left-handed β solenoid. Six β-strands form a two-rung (black and grey) triangular hydrophobic core. (**C**) Polar, hydrophobic, negatively charged and positively charged residues are coloured green, white, red and blue, respectively. (**D**) In α-SC3, K229, D230, R232, E234 and E235 in the first rung, and E265, T266, V268, K270 and G271 in the second rung, of HET-s were replaced by Q62, V63, N65, G67 and G68 of α-syn to model a fold in fibrillar α-syn with PDB ID 2N0A.^12^ (**E**) In α-SC6, R232, E234, (E235) and R236 in the first rung, and V268, K270, G271 and E272 in the second rung, of HET-s were replaced by K80, V82, E83 and G84 of α-syn to model a fold in fibrillar α-syn with PDB ID 6H6B.^13^ (**F**) In α-SC8, R225, S227 and K229 in the first rung, and T261, S263 and E265 in the second rung, of HET-s were replaced by K60, Q62 and T64 of α-syn to model a fold in fibrillar α-syn with PDB ID 6H6B. (**G**) In α-SC9, S227, K229, D230 and R232 in the first rung, and S263, E265, T266 and V268 in the second rung, of HET-s were replaced by K80, V82, E83 and A85 of α-syn to model a fold in fibrillar α-syn with PDB ID 6H6B.

The C-terminal residues 218–289 of HET-s are unstructured in solution and can form self-propagating fibrils that regulate heterokaryon incompatibility.^17^ Fibrillar HET-s(218–289) (Fig. 1A) consists of four β-strands that form two windings of a left-handed β-solenoid (Fig. 1B and C).^18,19^ Based on two structures of synthetic α-syn fibrils solved by solid-state NMR^12^ and cryo-EM,^13^ we introduced selected amino acid residues in human α-syn, which form distinct conformational surface epitopes only present in the fibrillar state (Fig. 1D–G) into HET-s(218–289) by mutagenesis to obtain the four vaccine candidates α-SC3 (Fig. 1D), α-SC6 (Fig. 1E), α-SC8 (Fig. 1F) and α-SC9 (Fig. 1G). Transmission electron microscopy revealed that purified proteins from all four vaccine candidates formed fibrils (Supplementary Fig. 1). To ensure that modified HET-s(218–289) fibrils bearing conformational surface epitopes of α-syn fibrils are safe and would not seed aggregation of α-syn in animals or humans after vaccination due to a partial similarity to α-syn fibrils, we assessed the ability of each vaccine candidate to seed aggregation of monomeric α-syn in a real-time quaking induced conversion (RT-QuIC) assay. In contrast to synthetic *N*-acetylated human α-syn fibrils (Supplementary Fig. 2), none of the four HET-s-derived fibrils induced aggregation of monomeric α-syn (Supplementary Fig. 3).^20^ We also evaluated the ability of the four vaccine candidates to seed α-syn aggregation in HEK293T cells expressing human α-syn with the familial A53T mutation fused to enhanced yellow fluorescent protein (α-synA53T-YFP; Supplementary Fig. 4).^21^ In this cell assay, transfection with synthetic human α-syn fibrils (Supplementary Fig. 2) seeded aggregation of α-syn A53T-YFP, but transfection with either one of the four vaccine candidates did not. The inability to seed aggregation of monomeric α-syn in the RT-QuIC assay and in cells suggested that the four vaccine candidates would be safe to use in mice overexpressing human α-syn without inadvertently inducing disease by seeding the aggregation of α-syn.

### α-Syn fibrils for vaccine candidate development and challenge are structurally nearly identical

**Figure 2:**
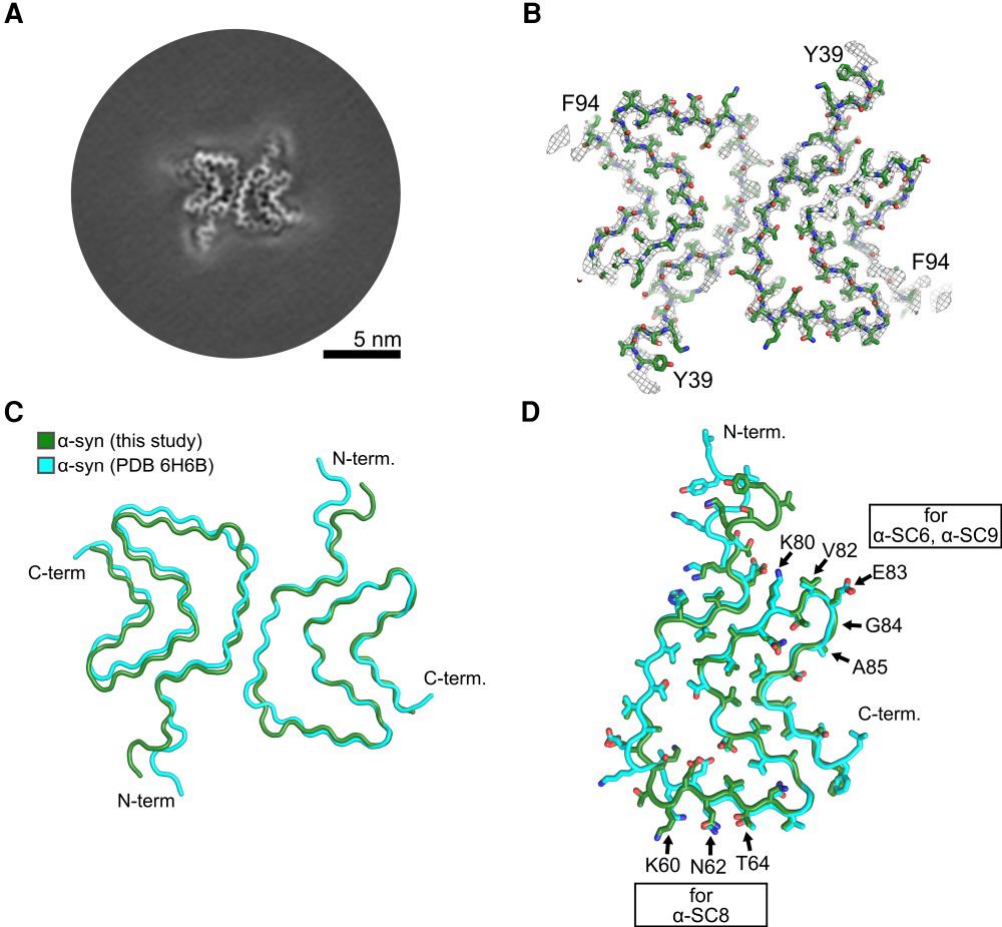
**Cryo-EM analysis shows a high similarity between α-synuclein (α-syn) fibrils used to develop vaccine candidates and those used to challenge TgM83^+/−^ mice (Protein Data Bank ID 8OQ1)**. (**A**) A cross-section through the reconstructed density map. (**B**) Superposition of the final density map (grey mesh) and the atomic model (green sticks). The labels indicate the N- and C-terminal residues. (**C**) Superposition of two opposing subunits or only a single chain (**D**) extracted from the atomic model of the *N*-acetylated human α-syn fibril and a previously determined wild-type α-syn fibril (shown in green) with Protein Data Bank (PDB) ID 6H6B (shown in cyan). Residues selected to model the vaccine candidates α-SC6, α-SC8 and α-SC9 are labelled.

Cryo-EM analysis of the full-length *N*-acetylated human wild-type α-syn fibrils used in our study to challenge TgM83^+/−^ mice revealed fibrils [Protein Data Bank (PDB) ID 8OQ1] that were structurally nearly identical to fibrils formed by non-acetylated and C-terminally truncated α-syn (PDB ID 6H6B; Fig. 2, Supplementary Fig. 5 and Supplementary Tables 1 and 2). Since the 6H6B fibril was considered in the design of vaccine candidates α-SC6, α-SC8 and α-SC9, the similarity between the α-syn fibrils used in this study and those used in the design of three of our vaccine candidates (PDB ID 6H6B) suggested that they could induce protective immunity in TgM83^+/−^ mice. Furthermore, structural similarities between the grafted conformational epitopes used in our vaccine candidates (Fig. 1D–G) and those present in *ex vivo* fibrils from patients with synucleinopathies also suggested that immunization with these vaccines could induce antibodies able to recognize patient brain homogenates (Supplementary Fig. 6).^14,15^

### Vaccination prolongs survival in brain-first and body-first mouse models of Parkinson’s disease

**Figure 3:**
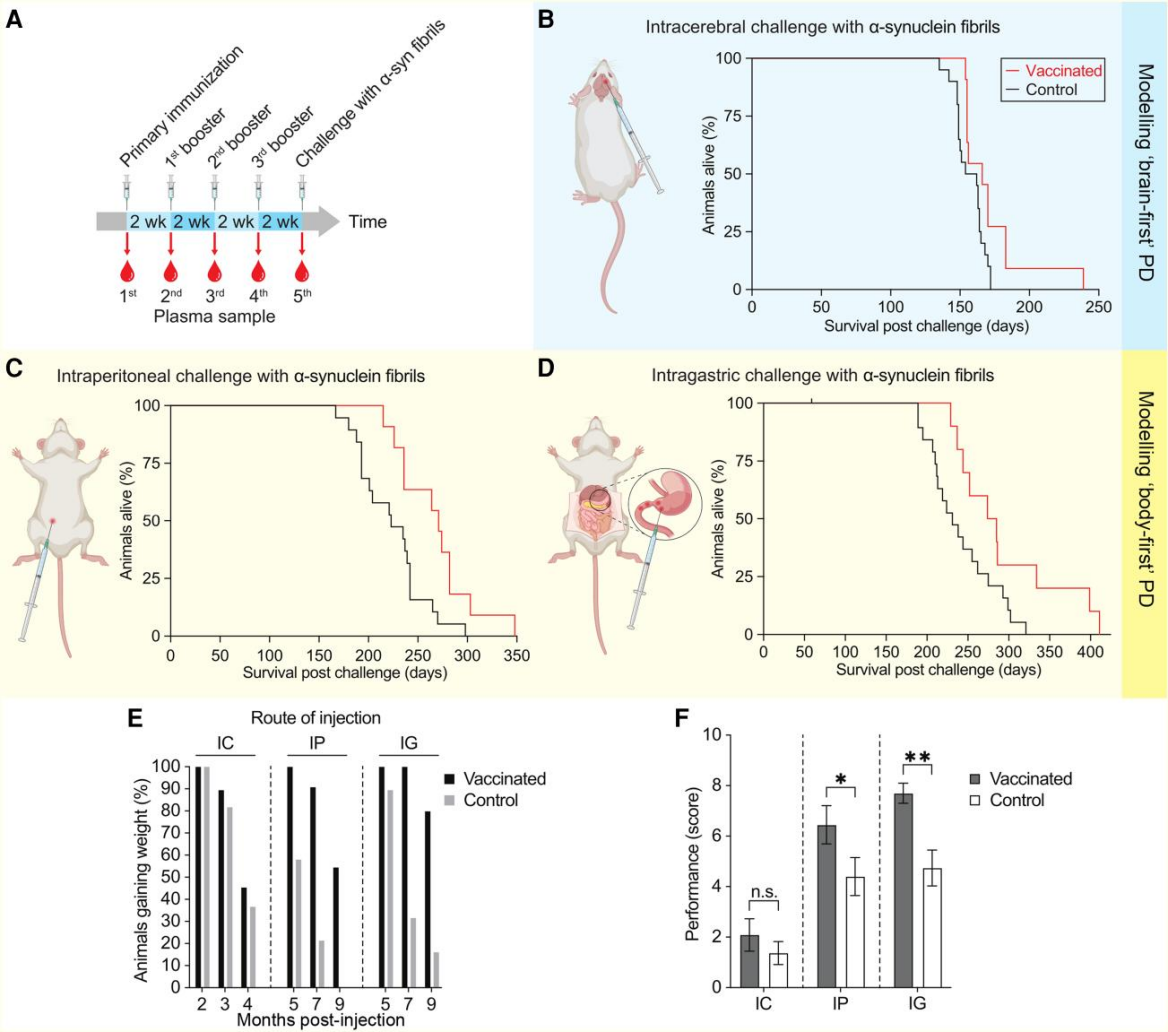
**Vaccination prolongs the survival of challenged TgM83^+/−^ mice and protects against motor dysfunction.** (**A**) TgM83^+/−^ mice were immunized intraperitoneally with a mixture of four vaccine candidates every 2 weeks for a total of four doses. To monitor the immune response, plasma was collected once before and every 2 weeks after administration of the quadrivalent vaccine. (**B**) TgM83^+/−^ mice were intracerebrally injected with α-synuclein (α-syn) fibrils to induce a synucleinopathy starting in the brain to model brain-first Parkinson’s disease (PD). Immunization significantly (*P* < 0.05) prolonged median survival from 154 days in unimmunized control mice (*n* = 20) to 166 days in fully vaccinated mice (*n* = 11). (**C** and **D**) To model body-first PD, mice were injected with α-syn fibrils into (**C**) the peritoneum or (**D**) the intestinal wall. (**C**) In intraperitoneally challenged mice, vaccination significantly (*P* < 0.01) extended median survival from 223 days in non-immunized control mice (*n* = 19) to 271 days in fully immunized mice (*n* = 11). (**D**) In mice injected with α-syn fibrils into the intestinal wall, vaccination significantly (*P* < 0.05) prolonged median survival from 231 days in non-immunized control mice (*n* = 20) to 280 days in fully immunized mice (*n* = 10). Survival was analysed using Kaplan–Meier curves and the log-rank (Mantel–Cox) test. (**E**) TgM83^+/−^ mice challenged with α-syn fibrils initially gained weight to a maximum before they began to lose weight and develop neurological disease. In all three models of brain-first and body-first PD, immunized animals gained weight for longer periods of time than non-immunized animals. For mice injected intracerebrally with α-syn fibrils, the percentage of animals gaining weight is shown at 2, 3 and 4 months post challenge, and for animals injected intraperitoneally or into the intestinal wall at 5, 7 and 9 months post challenge. (**F**) The motor performance of immunized and non-immunized TgM83^+/−^ mice was assessed at different time points after challenge with α-syn fibrils using the rotarod, grip strength and the pole test assays. In animals injected intracerebrally with α-syn fibrils, the cumulative performance score of immunized animals was higher than that of non-immunized animals but did not reach significance at 5 months post challenge. For animals injected intraperitoneally or into the intestinal wall with α-syn fibrils, the cumulative performance score of immunized animals was significantly higher than that of non-immunized animals at 7 months post challenge. Bars represent the mean ± SEM. Welch’s *t*-test. *P < 0.05, **P < 0.01. IC = intracerebral; IG = into the gut wall; IP = intraperitoneal.

Hemizygous TgM83^+/−^ mice express human α-syn with the familial A53T mutation but do not naturally develop any neuropathology or disease for up to 650 days.^10,22^ Injection of patient-derived and synthetic α-syn fibrils induces a PD-like neuropathology and disease in these mice with variable incubation times depending on the dose and the route of injection.^10,23^ To determine whether vaccination with a quadrivalent mixture of α-SC3, α-SC6, α-SC8 and α-SC9 fibrils could induce immunity to misfolded α-syn and protect against the neuropathology and disease it causes, we injected adult TgM83^+/−^ mice intraperitoneally with the quadrivalent mixture every 2 weeks for a total of four times (Fig. 3A). To monitor the immune response of TgM83^+/−^ mice, we collected plasma once before and every 2 weeks after the vaccine was administered and stored it for later analysis (Fig. 3A).

Recent evidence from human and animal studies suggests that PD patients can be subtyped into those in whom the α-syn pathology associated with prodromal PD first develops in either the CNS or the ENS, referred to as brain-first and body-first PD, respectively.^24^ Subsequently, α-syn pathology may spread in a prion-like manner from the CNS to the peripheral nervous system in brain-first PD or from the ENS to the CNS in body-first PD, resulting in a late stage disease that is very similar in both PD subtypes. To model brain-first PD, we intracerebrally injected fully vaccinated TgM83^+/−^ mice with synthetic α-syn fibrils (Fig. 2 and Supplementary Fig. 5) to induce synucleinopathy starting in the brain (Fig. 3B). Immunization significantly (*P* < 0.05) prolonged median survival from 154 days in unvaccinated control mice (*n* = 20) to 166 days (8%) in vaccinated mice. To model body-first PD, we injected vaccinated TgM83^+/−^ mice with α-syn fibrils either into the peritoneum (Fig. 3C) or into the wall of the stomach and pylorus (Fig. 3D). In intraperitoneally challenged mice, immunization significantly (*P* < 0.01) extended median survival from 223 days in unvaccinated control mice to 271 days (22%) in vaccinated mice. In mice injected with α-syn fibrils into the gut wall, immunization significantly (*P* < 0.05) extended median survival from 231 days in unvaccinated control mice to 280 days (21%) in vaccinated mice. In general, mice injected with α-syn fibrils into the peritoneum or gut wall died later than mice injected into the brain because of the additional time it takes for pathologic α-syn to spread from the ENS to the CNS. We confirmed neurological disease in diseased mice (Supplementary Fig. 7) either by immunohistochemistry or immunofluorescence staining for deposits of pathologic α-syn in the brainstem, which was accompanied by microgliosis and astrogliosis, or by biochemistry for accumulation of pathological α-syn aggregates in brain homogenates. In conclusion, vaccination prolonged the median survival of TgM83^+/−^ mice and delayed the onset of PD-like neuropathological mechanisms in the CNS.

### Vaccination protects against weight loss and motor performance deficits

We monitored TgM83^+/−^ mice challenged with α-syn fibrils for weight loss and decline in motor performance, both of which are early surrogate markers of neurological disease. Both immunized and non-immunized TgM83^+/−^ mice challenged with α-syn fibrils initially gained weight before losing weight (Supplementary Fig. 8) and developing neurological disease. In all three models of brain-first and body-first PD, immunized mice gained weight for longer periods of time than non-immunized mice (Fig. 3E). All TgM83^+/−^ mice injected with α-syn fibrils into the brain were still gaining weight 2 months after challenge. At 3 months post challenge, this decreased to 90% for immunized and 82% for non-immunized mice. At 4 months post challenge, only 46% of immunized and 37% of non-immunized mice were still gaining weight. All TgM83^+/−^ mice injected with α-syn fibrils into the peritoneum or intestinal wall still gained weight at 5 months after challenge. For intraperitoneally challenged mice, this decreased to 91% for immunized and 58% for non-immunized mice at 7 months post challenge. Among these, only 55% of the immunized and 21% of the non-immunized mice were still gaining weight at 9 months post challenge. In mice injected with α-syn fibrils into the intestinal wall, 100% of the immunized mice and 90% of the non-immunized still gained weight 7 months after challenge. At 9 months post challenge, this was reduced to 80% in immunized mice and 32% in non-immunized. Overall, we observed a slower weight loss in all immunized animals after challenge with α-syn fibrils, regardless of the route of challenge. Since the disease progressed more rapidly in animals challenged intracerebrally than in those challenged peripherally, also weight loss progressed more quickly in animals challenged intracerebrally than in those challenged peripherally.

We evaluated the behavioural performance of immunized and non-immunized TgM83^+/−^ mice at different time points after challenge with α-syn fibrils using the rotarod, grip strength, and pole-test assays and used them to calculate a cumulative performance score (Fig. 3F). In animals challenged intracerebrally with α-syn fibrils, the cumulative performance score of immunized animals was higher (2.09 ± 2.12) than that of non-immunized animals (1.37 ± 1.98) but did not reach significance at 5 months post-challenge because many animals in both immunized and non-immunized cohorts were already ill at that time. If we had selected an earlier time point, such as 4 months after the intracerebral challenge, we may have been able to detect a difference in motor performance. In animals injected intraperitoneally with α-syn fibrils, immunized animals had a significantly (*P* < 0.05) higher cumulative performance score (6.45 ± 2.50) than non-immunized animals (4.40 ± 3.35) at 7 months after challenge with α-syn fibrils. In animals injected with α-syn fibrils into the intestinal wall, the cumulative performance score of immunized animals (7.70 ± 1.25) was also significantly higher (*P* < 0.001) than that of non-immunized animals (4.74 ± 3.11) 7 months after challenge with α-syn fibrils. Overall, immunized animals showed better motor function for longer periods of time after challenge with α-syn fibrils.

### Vaccination induces antibodies that recognize pathologic α-syn

**Figure 4:**
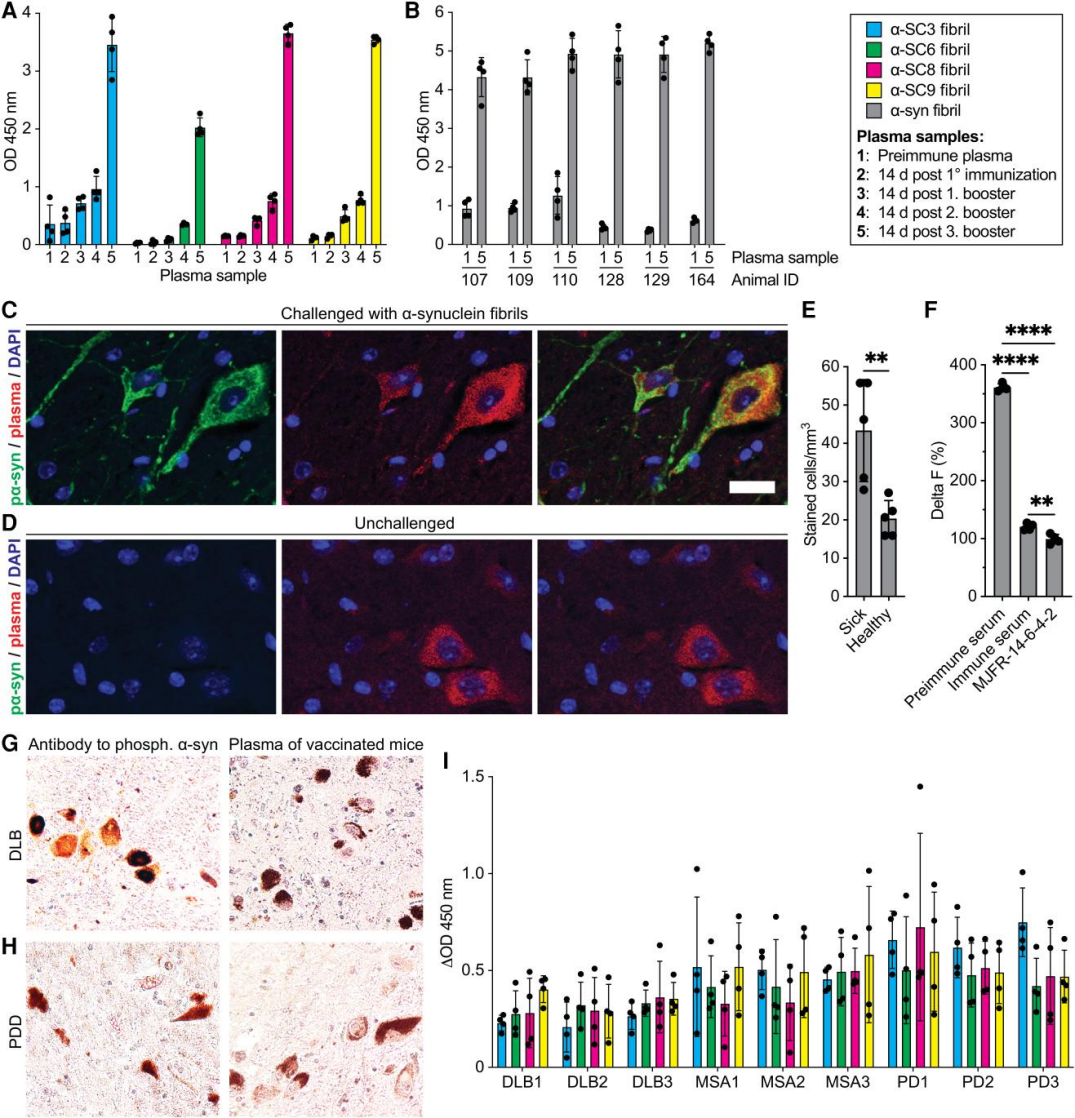
**Vaccination of TgM83^+/−^ mice with HET-s-derived vaccine candidates induces antibodies targeting pathologic α-synuclein.** (**A**) The immune response to each of the four fungal fibrils increased progressively with each booster vaccination. d = days; OD = optical density. (**B**) Antibodies from fully vaccinated mice recognized α-synuclein (α-syn) fibrils better than antibodies from unvaccinated mice. (**C**) Immunofluorescence staining of brainstem sections from diseased TgM83^+/−^ mice with an antibody against α-syn phosphorylated at serine 129 (pα-syn, green) revealed somatic and neuritic deposits of pathologic α-syn that largely colocalized with deposits detected by antibodies from immunized mice (red) as shown in the merged image on the *right*. (**D**) In brainstem sections from healthy control mice, pα-syn (green) was not detected, but antibodies from immunized mice (red) detected some monomeric α-syn. Cell nuclei were stained with DAPI. The scale bar represents 20 µm. (**E**) The staining results indicate a significantly (*P* < 0.01) higher number of stained cells in brainstem sections of sick mice compared with healthy mice. (**F**) Time-resolved fluorescence resonance energy transfer (FRET) quantification was used to measure oligomeric and aggregated α-syn in brain homogenates of sick mice after immunoprecipitation with plasma antibodies coupled to magnetic beads. The results showed that vaccinated mice had α-syn recognizing antibodies in their plasma in contrast to non-vaccinated mice. The MJFR-14-6-4-2 antibody, which recognizes α-syn aggregates, was used as a positive control. The Delta F (%) value is calculated by dividing the difference between the ratio of the sample and the ratio of the negative control by the ratio of the negative control, and then multiplying the result by 100. (**G** and **H**) Immunohistochemical staining was performed on human brain tissue sections from a patient with dementia with Lewy bodies (DLB) (**G**) and one with Parkinson’s disease with dementia (PDD) (**H**). The results showed that the pSyn#64 antibody to α-syn phosphorylated at serine 129 (*left*) and plasma of fully vaccinated mice (*right*) were both able to detect pathologic α-syn. (**I**) In a competitive ELISA, antibodies from fully immunized animals (*n* = 4) recognized brain homogenates from patients with DLB (*n* = 3), multiple system atrophy (MSA, *n* = 3) or Parkinson’s disease (*n* = 3) better than brain homogenates from healthy controls (*n* = 5). Bars represent mean ± standard deviation. **P < 0.01, ****P < 0.0001.

Analysis of plasma collected from immunized mice by ELISA showed that the primary and three subsequent booster vaccinations had induced a progressive increase in antibody titres recognizing the four vaccine candidates, reaching a 10- to 70-fold increase in detection in fully immunized animals (Fig. 4A and Supplementary Fig. 9). Importantly, immunization with the vaccine candidates also increased immune recognition of α-syn fibrils (50 nM monomer equivalent) (Fig. 4B). Antibodies from fully immunized mice also recognized deposits of pathologic α-syn in the CNS of diseased mice and, to a lesser extent, monomeric α-syn in the CNS of healthy control animals, as detected by immunofluorescence staining (Fig. 4D and E). To demonstrate the presence of antibodies that recognize α-syn in the plasma of fully vaccinated mice, we immunoprecipitated α-syn from the brain homogenate of a sick mouse using preimmune serum, serum from fully vaccinated mice (immune serum) and the MJFF-14-6-4-2 antibody to misfolded α-syn coupled to protein G-coated magnetic beads. We then measured the amount of α-syn aggregates in the cleared brain homogenate using time-resolved fluorescence resonance energy transfer (FRET) analysis (Fig. 4F). Immunohistochemical staining was performed on sections of the substantia nigra from a patient with dementia with Lewy bodies (DLB) (Fig. 4G) and one with PD with dementia (Fig. 4H and Supplementary Table 3). The plasma of fully vaccinated mice was found to contain antibodies detecting pathologic α-syn. In a competitive ELISA, antibodies from fully immunized animals also recognized pathologic α-syn in brain homogenates from patients (Supplementary Table 4) with DLB (*n* = 3), MSA (*n* = 3) or PD (*n* = 3) when tested against brain homogenates from five healthy controls (Fig. 4I and Supplementary Fig. 10). While all four vaccine candidates induced antibodies that recognized patient brain homogenates of all three synucleinopathies better than the healthy control brain homogenates, the four vaccine candidates showed different efficacy against different disease-derived fibrils. For example, α-SC3 and α-SC8 induced a significantly (*P* < 0.05) stronger antibody response for PD than for MSA or DLB compared to α-SC6 and α-SC9 (Supplementary Fig. 11).

## Discussion

The identification of the structures of α-syn fibrils has enabled us to engineer novel conformation-specific vaccines by epitope grafting onto a carrier molecule that can elicit a targeted immune response to pathogenic α-syn assemblies with a significant delay of disease onset times in one mouse model of brain-first PD and two mouse models of body-first PD. Previous vaccination strategies that have entered clinical trials have used modified, 8–12 amino acid C-terminal peptide fragments of α-syn to induce immune responses directed against its pathological form.^25-30^ The quadrivalent vaccine developed here induced an antibody response that not only recognized synthetic α-syn fibrils and pathologic α-syn from TgM83^+/−^ mice but also from patients suggesting that it may also provide protection against synucleinopathies in humans. Considering that PD, LBD and MSA are primarily age-related diseases, even relatively short extensions of disease onset times would translate to significant health benefits and an improved quality of life for the ageing population. The vaccine candidates designed and tested here were modelled after a few conformational epitopes present on only two structures of synthetic α-syn fibrils, and yet provided considerable protection from disease. We only used a limited selection of possible conformational epitopes from a larger conformational space to design the vaccine candidates tested here. It is conceivable that other conformational epitopes could confer better immunity and protection from disease. Also, the recent availability of *ex vivo* structures of α-syn fibrils isolated from patients with synucleinopathies should enable the design of disease-specific and, thus, more potent vaccine candidates.^14,15^ There may be an overlap of conformational epitopes between these vaccines and those found in α-syn fibrils in patients with PD, DLB and MSA. This overlap may explain why antibodies from immunized mice recognize pathologic α-syn in patient brains and could also explain differences in the induced antibody response (Supplementary Figs 6 and 11).

Importantly, conformational epitope engineering is suitable for the development of vaccines against a broad spectrum of pathogenic protein structures and amyloids, such as amyloid beta and tau in Alzheimer’s disease, islet amyloid polypeptide (IAPP) in type II diabetes and others causing protein misfolding diseases. Lastly, fibrillar vaccines are structurally stable at room temperature for long periods of time, facilitating their storage, transport and distribution.

## Supplementary Material

awae061_Supplementary_Data

## Data Availability

All data are available in the manuscript or the Supplementary material. The cryo-EM map has been deposited in the Electron Microscopy Data bank (EMDB) under the accession number EMD-17111 and the corresponding atomic model in the Protein Data Bank (PDB) under the accession number 8OQI. Additional data will be available from the corresponding authors upon reasonable request. Materials can be made available with a material transfer agreement.
