# Supplementary material for: Vaccination with structurally adapted fungal protein fibrils induces immunity to Parkinson’s disease
Source: Brain. 2024 Mar 1;147(5):1644–52. doi: 10.1093/brain/awae061 (PMC11068327; doi:10.1093/brain/awae061)
Supplement: awae061_Supplementary_Data [file awae061_supplementary_data.pdf]

Supplementary Material

to

Vaccination with structurally adapted fungal protein fibrils induces immunity to Parkinson's disease

Verena Pesch,[1,†] José Miguel Flores-Fernandez,[2,†,¶] Sara Reithofer,[1,†] Liang Ma,[1,†] Pelin Özdüzenciler,[1] Yannick Busch,[1] Aishwarya Sriraman,[2] YongLiang Wang,[2,#] Sara Amidian,[2] Chiara V. M. Kroepel,[1] Laura Müller, [1] Yi Lien,[1] Olivia Rudtke,[1] Benedikt Frieg,[1] Gunnar F. Schröder,[1,3] Holger Wille,[2,4] and Gültekin Tamgüney[1,5]

Materials and methods

Animals

Hemizygous B6;C3-Tg(*Prnp*-SNCA*A53T)83Vle/J mice (TgM83$^{+/-}$ mice) were obtained from The Jackson Laboratory and bred with wild-type C57BL/6J mice to produce hemizygous offspring. The presence of transgenes encoding human α-syn with the familial A53T mutation was determined by real-time PCR. Cohort sizes were determined a priori to allow for the detection of significant differences in motor function and survival. No animals were excluded during the experiment. Animals were randomly allocated to treatment groups based on birth and availability. Animals in different cohorts were caged independently of the treatment and all held at the same location. V.P, S.R., L.M., and G.T. were aware of the group allocation at the different stages. All studies involving animals were approved by the animal protection committee of the North Rhine-Westphalia State Environment Agency (LANUV). All applicable national and institutional guidelines for the care and use of animals were followed.

Preparation of HET-s-derived fibrils

Constructs for the four vaccine candidates were designed using UCSF ChimeraX software. DNA encoding the four vaccine candidates was purchased (Bio Basic) and cloned into a pET-21a vector (Novagen) for expression in *E. coli* BL21(DE3). Starter cultures were grown in 25 mL of 2YT media at 37 °C and 250 rpm overnight, and used to inoculate 500 mL of 2YT media with 100 µg/mL ampicillin, which was grown to an optical density of 0.8 at 600 nm. The culture was cooled down to 25 °C, and induced with 1 mM isopropyl-beta-D-1-thiogalactopyranoside (IPTG) and grown overnight at 37 °C and 250 rpm. Cells were harvested at 5,000 rpm and 4 °C for 25 min, and frozen for at least 30 min. The pellet was resuspended in 20 mL IB buffer (100 mM Tris-HCl, pH 8.0) with 0.5 % Triton X-100, 1 mg/mL lysozyme (Sigma-Aldrich), and 1× complete EDTA-free protease inhibitor cocktail (Roche). After incubation at room temperature for 30 min, the lysed cells were sonicated (Sonifier 250; Branson Ultrasonics) for 5 cycles (1 min on and 1 min off) at an output voltage of 50 with a 50% duty cycle at 4 °C. Subsequently, 3 U/mL benzonase (Merck) for each milliliter of the original culture was added, and the

homogenate was incubated for 20 min, and then centrifuged at 11,000 rpm and 4 °C for 30 min. The supernatant was discarded, and pellets containing the expressed protein as inclusion bodies were frozen for at least 30 min. The pellets were resuspended in IB buffer containing 0.5% Triton X-100, 1 mg/mL lysozyme, and 1× complete EDTA-free protease inhibitor cocktail, and were then incubated for 20 min at RT. After another sonication and centrifugation step as described above, the process was repeated twice and the homogenate centrifuged at 11,000 rpm, 4 °C for 30 min. Finally, the pellet containing purified inclusion bodies was resuspended in IB buffer and stored at −20 °C. Inclusion bodies of recombinant proteins were solubilized in 6 M guanidine hydrochloride, 20 mM sodium phosphate, 0.5 M NaCl at pH 8.0, and stirred at room temperature for 45 min. The homogenate was clarified by ultracentrifugation at 45,000 rpm for 35 min at 4 °C. Recombinant protein was purified under denaturing conditions by affinity chromatography using a BioLogic DuoFlow chromatography system (Bio-Rad Laboratories). A HisTrap HP column (GE Healthcare Life Sciences) was equilibrated with equilibrium buffer (8 M Urea, 20 mM sodium phosphate, 0.5 M NaCl, 10 mM imidazole, pH 8.0) until the absorbance at 280 nm was stable. The column was loaded with the clarified lysate and then washed with equilibrium buffer until the absorbance at 280 nm was stable again. Bound protein was eluted using a linear gradient of 10 mM to 500 mM imidazole in equilibrium buffer. Peaks containing recombinant protein were automatically collected with a BioFrac fraction collector (Bio-Rad Laboratories) when the absorbance at 280 nm was >0.05. Subsequently, the buffer was exchanged to 175 mM acetic acid at pH 2.8 using a HiTrap desalting column (GE Healthcare Life Sciences). The purified, desalted, and denatured protein was fibrillized by increasing the pH to 7.5 with 3 M Tris base (pH 13.0) and stirring at 600 rpm at room temperature for 5 d.

Vaccinations and plasma collection

At 6–8 weeks of age, mice were anesthetized with 2–3% isoflurane and intraperitoneally injected with 200 µL of 100 µg antigen, composed of equal amounts of α-SC3, α-SC6, α-SC8, and α-SC9 fibrils, diluted 1:1 in alum (Alhydrogel adjuvant 2%, InvivoGen) using a 26-gauge needle. Each animal received four vaccine injections, each two weeks apart. Immediately before each vaccine injection and two weeks after the final, 50 µL of blood was collected from the tail vein of each

mouse and diluted 1:1 with 5% sodium citrate. Blood samples were centrifuged at 500 × g for 10 min at 4 °C, and the resulting plasma collected and stored at −80 °C.

Preparation of α-syn monomers and fibrils

N-terminally acetylated human wild-type α-syn was expressed in *E. coli* BL21(DE3) carrying the pT7 vector for codon-optimized α-syn and the pNatB vector for the N-terminal acetyltransferase B complex from *Schizosaccharomyces pombe*. The human K23Q mutant of α-syn was expressed in *E. coli* BL21(DE3) from the pET-28a vector. Bacteria were cultured in 120 mL lysogeny broth (LB) medium with 100 µg/mL ampicillin and 34 µg/mL chloramphenicol at 37 °C and 120 rpm overnight. The next day, the optical density was measured at 600 nm and the culture was diluted to an optical density of 0.1 in 1 L of LB medium. The culture was then incubated with 100 µg/mL ampicillin and 34 µg/mL chloramphenicol at 37 °C until the optical density reached 1.0–1.2. Expression was induced with 1 mM of IPTG. After 4.5 h, the cells were pelleted at 5000 × g and 4 °C. The pellets were resuspended in 20 mM Tris (pH 8.0) containing a protease inhibitor (Roche) and were boiled for 2 × 15 min, then centrifuged at 20,000 × g and 4 °C for 30 min. Ammonium precipitation was performed with 0.45 g/mL of $(NH_4)_2SO_4$ crystals, which were added to the supernatant over 5 min and stirred for 15 min. The centrifugation was repeated and the pellet was resuspended in 50 mL of 20 mM Tris-HCl (pH 8.0). α-Syn was purified using the HiPrep QFF 16/10 anion exchange chromatography column and a linear gradient from 20 mM Tris-HCl (pH 8.0) binding buffer to 1 M NaCl in 20 mM Tris-HCl (pH 8.0) elution buffer on an ÄKTA pure chromatography system (GE Healthcare). The ammonium precipitation was repeated and the pellet was resuspended in 50 mM Tris-HCl (pH 7.2) and purified using a HiLoad 16/60 Superdex 75 pg size exclusion column (Cytiva) over 1.5 column volumes. NaCl was added to obtain 50 mM Tris-HCl and 150 mM NaCl. The protein was concentrated to 5 mg/mL using a vivaspin concentrator (Sartorius). The α-syn monomer was fibrillized by incubation at 37 °C and 1000 rpm on a Thermomixer (Eppendorf) for 7 d. Fibrils were sonicated by four 15 s sonification steps with 2 min pauses between each step using a Sonoplus Mini20 (Bandelin) and an MS 1.5 microtip.

Cryo-EM grid preparation and imaging of N-acetylated human wild-type α-syn fibrils

For cryo-EM grid preparation, 3 µL of the wild-type α-syn fibril solution was applied to freshly glow-discharged R1.2/1.3 holey carbon film grids (Quantifoil). After the grids were blotted for 5 s, the grids were flash frozen in liquid ethane using a Mark IV Vitrobot (Thermo Fisher Scientific). Cryo-EM data sets were collected on a Titan Krios G4 transmission electron microscope (Thermo Fisher Scientific) operated at 300 keV accelerating voltage and a nominal magnification of 81,000 × using a K3 direct electron detector (Gatan) in superresolution counting mode, corresponding to a calibrated pixel size of 1.06 Å (Table S2). A total of 7,316 movies were collected EPU (Thermo Fisher Scientific). Movies were recorded over 30 frames accumulating a total dose of ~30 $e^{-}/Å^{2}$. The range of defocus values collected spans from −0.5 µm to −2.5 µm. Collected movies were motion-corrected and dose-weighted on the fly using Warp.

Helical reconstruction of N-acetylated human wild-type α-syn fibrils

α-Syn fibrils were reconstructed using RELION-3.1, following the helical reconstruction scheme. Firstly, the estimation of contrast transfer function parameters for each motion-corrected micrograph was performed using CTFFIND4. Next, filament picking was done using crYOLO. For 2D classification, we extracted 1,590,869 particle segments using a box size of 600 pix downscaled to 200 pix and an inter-box distance of 13 pix (1.06 Å/pix). For 3D classification, the classified segments after 2D classification were (re-)extracted using a box size of 250 pix and without downscaling. Starting from a featureless cylinder filtered to 60 Å, several rounds of refinements were performed while progressively increasing the reference model's resolution. The helical rise was initially set to 4.75 Å and the twist was estimated from the micrographs, assuming a left-handed twist. Once the β-strands were separated along the helical axis, we optimized the helical parameters (final parameters are reported in Supplementary Table 2). Finally, we performed a gold-standard 3D auto-refinement. Standard RELION post-processing with a soft-edged solvent mask that includes the central 10 % of the box height yielded post-processed maps (B-factors are reported in Supplementary Table 2). The resolution was estimated

from the value of the FSC curve for two independently refined half-maps at 0.143 (Fig. 5). The optimized helical geometry was then applied to the post-processed maps yielding the final maps used for model building.

Atomic model building and refinement of N-acetylated human wild-type α-syn fibrils

One protein chain was extracted from PDB-ID 6H6B of wild-type α-syn and optimized into the final cryo-EM map using Coot.[1] Refinement in real space was conducted using PHENIX and Coot in an iterative manner. The resulting model was validated with MolProbity.

Challenge of TgM83$^{+/-}$ mice with α-syn fibrils

For intracerebral challenge, TgM83$^{+/-}$ mice received a subcutaneous injection of Carprofen (5mg/kg) for analgesia, were anesthetized with isoflurane, and received a stereotactic injection of 10 µg of sonicated α-syn fibrils in 4 µL phosphate-buffered saline (PBS, Sigma) into the right striatum (coordinates: +0.2 mm relative to the bregma, −2.0 mm relative to the midline, and 2.6 mm below the dura). A flow rate of 1 µL/min was used for stereotactic delivery, and the needle was left in place for an additional minute before being slowly retracted.

For intraperitoneal challenge, 50 µg of sonicated α-syn fibrils in 20 µL PBS were injected into the peritoneum of mice anesthetized with isoflurane using a 30-gauge disposable hypodermic needle.

For injections of α-syn fibrils into the gut wall, aseptic laparotomy was performed. For analgesia, animals received a subcutaneous injection of buprenorphine (0.05 mg/kg, Bayer) and were then anesthetized with isoflurane. The abdomen was shaved, disinfected, and opened with a small incision using a scalpel. α-Syn fibrils were injected into the wall of the pylorus and duodenum at four points, spaced 0.5 cm apart, using a 10 µL Hamilton syringe. Each injection site was injected with 6.25 µg (2.5 µL) of α-syn fibrils. The abdominal wall was then sutured and the skin closed with wound clips that were removed two weeks later. After surgery, the animals received

a single subcutaneous dose of Carprofen (5 mg/kg) and were supplied with a mix of metamizole (0.5 mg/mL, WDT) and 10% (w/v) sacharose in their drinking water for 3 days.

Animals were monitored daily for general health and three times a week for signs of neurological disease, including but not limited to reduced grooming, ataxia, tremor, bradykinesia, akinesia, lethargy, circling, tail rigidity, paraparesis, paralysis, kyphosis, and others. Body weight was recorded weekly. Diseased mice were euthanized with ketamine/xylazine and underwent transcardial perfusion with PBS followed by 4% formalin (Sigma) in PBS. The brains were then either dissected and fixed overnight with 4% formalin in PBS overnight for immunohistochemistry or snap-frozen on dry ice for later biochemical analysis. GraphPad Prism 9 was used for statistical analysis.

Immunoprecipitations

To test the specificity of the vaccination-induced antibodies for α-syn, immunoprecipitation with magnetic beads was performed. Briefly, 50 µL of plasma was incubated with 16.7 µL of Protein G-coupled Dynabeads (Invitrogen) for 1 h with rotation at room temperature. As a positive control, the beads were incubated in a 0.0135 µg/µL solution of the conformation-specific anti-α-syn aggregate antibody MJFR-14-6-4-2 (abcam) in PBS with 0.02% Tween 20. The beads were washed three times with 50 µL of PBS. The 5 µg total protein beads were suspended in 50 µL samples prepared from 10% (w/v) brain homogenates in 1×lysis buffer. The samples were incubated overnight with rotation at 4 °C. The quantity of remaining oligomers and aggregates of α-syn in the treated brain homogenates were quantified by time-resolved FRET.

Time-resolved FRET

The quantification of oligomers and aggregates of α-syn in brain homogenates was performed using a commercially available kit (Revvity) and time-resolved fluorescence resonance energy transfer (FRET). Briefly, 10-µL samples with 1 µg total protein were prepared from 10% (w/v) brain homogenates in 1×lysis buffer. Each sample was supplemented with 10 µL of a pre-mixed antibody solution containing anti-h-α-Synuclein-d2 (acceptor) and anti-h-α-Synuclein-Tb-

Cryptate (donor). A negative control, Cryptate control, and buffer control were prepared according to the manufacturer's instructions. The final mix, containing the sample and antibody, as well as the controls, was transferred to a HTRF 96-well low volume plate (Revvity), covered with a plate sealer, and incubated for 20 h at room temperature. Fluorescence emission was measured at 665 nm for FRET-dependent acceptor fluorescence and at 620 nm for FRET-independent donor fluorescence using a CLARIOstar microplate reader (BMG Labtech). The ratio of both fluorescence emission values, multiplied by 10,000, is directly proportional to the amount of human α-synuclein oligomers and aggregates in each sample. The Delta F (%) value is a measure of the signal-to-background ratio in the assay. It is calculated by dividing the difference between the ratio of the sample and the ratio of the negative control by the ratio of the negative control, and then multiplying the result by 100. The negative control is used as an internal control.

ELISA

Briefly, 96-well high-binding ELISA plates (Corning, Cat# 9018) were coated overnight with 100 µL/well of a 5 µg/mL solution of sonicated fibrils at 4 °C with gentle agitation. The plates were then washed twice with phosphate-buffered saline (PBS) with 0.1% Tween20 (PBST) and once with PBS, blocked with 200 µL/well blocking buffer (5% (w/v) milk powder in PBS), then washed again five times with PBST and once with PBS. After adding 100 µL of plasma to each well, the plates were incubated overnight at 4 °C. Plasma of one mouse collected at 5 different time points was tested at a 1:330,000 dilution in PBS. For comparing preimmune plasma with plasma collected 14 d after the third booster of nine mice, preimmune plasma was diluted 1:10,000 and plasma collected after the final booster 1:330,000 in PBS. For detection of α-syn fibrils, immune plasma was diluted 1:1000 in PBS. Following five washes with PBST and one with PBS, the plates were incubated for 2 h at room temperature with 100 µL/well of an anti-mouse horseradish peroxidase-linked secondary antibody (Invitrogen) at a 1:5000 dilution. After five washes with PBST and one with PBS, the plates were incubated with 100 µL/well TMB substrate solution (Pierce TMB Substrate Kit, Thermo Fisher Scientific). The color reaction was stopped after 50 min with 2 M sulfuric acid and the absorbance measured at 450 nm with a CLARIOstar microplate reader (BMG Labtech).

Competitive ELISA

A competitive ELISA protocol was used to measure recognition of α-syn aggregates in patient brain homogenates by plasma of fully immunized mice. A 96-well high-binding ELISA plate (Corning, Cat# 9018) was coated overnight with 100 µL/well of a 5 µg/mL solution of sonicated HET-s-derived fibrils at 4 °C with gentle agitation, then blocked with 3% bovine serum albumin (BSA) in PBS for 90 min and washed twice with PBST and once with PBS. On another 96-well low-binding cell culture plate, 75 µL/well of a 1:330,000 dilution of plasma was mixed with 66.67 µg/mL of brain homogenate (DLB, MSA, PD, or healthy control) and incubated overnight at 4 °C. Next, 100 µL/well of the mixture was added to the fibril-coated and blocked ELISA plates and incubated overnight at 4 °C. Following two washes with PBST and one with PBS, 100 µL/well of an anti-mouse horseradish peroxidase-linked secondary antibody was added at a 1:5000 dilution and incubated at room temperature for 2 h. After another four washes with PBST and one with PBS, 100 µL/well of TMB substrate solution was added. The color reaction was stopped after 30 min with 2 M sulfuric acid and absorbance measured at 450 nm with a CLARIOstar microplate reader.

Motor function analysis

Animals were tested at 2 and 5 months after intracerebral injections with α-syn fibrils, and at 3 and 7 months after intraperitoneal injections or injections into the wall of the gastrointestinal tract.

Motor function and coordination were evaluated with a rotarod test. Mice were placed on a horizontal and accelerating (4 to 40 rpm) rotarod treadmill (Ugo Basile) for a maximum of 300 s. The time an animal managed to run on the rotarod was recorded. Each animal was tested four times with a 5 min rest period between each trial on a particular test day, and the mean value of the last three trials was taken as an animal's performance on that day.

To measures grip strength, mice were given a grid attached to a grip strength meter (Ugo Basil) to grab with all four paws, and were then gently pulled by the tail until they released the rod. The

grip strength was measured three times with 15 min breaks between each test. The mean value of the three trials was taken as an animal's performance on that day.

Motor function and balance were tested with a pole test. Animals were placed facing away from the ground on top of a 75 cm high pole with a 9 mm diameter and wrapped in gauze. Once the animals turned around to face downwards, their time of descend was measured. Animals were tested three times. The assay was stopped if an animal took longer than 60 s. Animals falling or sliding down the pole were given a time of 60 s. The mean value of the three trials was taken as an animal's performance on that day.

The percentage of an animal's mean performance in each assay was calculated relative to its mean performance at an earlier time point. Performances in the rotarod and grip strength assays above 75% of an earlier measurement received a score of 1, above 81% a score of 2, above 93% a score of 3, and all else a score of 0. Performances in the pole test below 160% of an earlier measurement received a score of 1, below 130% a score of 2, below 110% a score of 3, and all else a score of 0. A final cumulative performance score (0–9) was calculated by summarizing the performance scores in the rotarod (0–3), grip strength (0–3), and pole test (0–3) assays. GraphPad Prism 9 was used for statistical analysis. Significance was assessed using an unpaired t test with Welch's correction for non-parametric data.

Immunofluorescence analysis

Paraffin-embedded tissues were cut into 8-µm-thick coronal sections, mounted on slides, deparaffinized, and rehydrated. Antigen retrieval was conducted as described for immunohistochemical analysis. The samples were washed with PBS at room temperature. Tissue autofluorescence was quenched by incubation in $CuSO_4$ for 90 min at room temperature. The slides were blocked in 20% (v/v) normal goat serum, 1% (v/v) BSA, and 0.5% (v/v) Triton X-100 in PBS for 1 h at room temperature. After blocking, the sections were incubated with a primary antibody in 1% (v/v) normal goat serum, 1% (v/v) BSA, and 0.25% Triton X-100 in PBS overnight at room temperature. After washing once with washing buffer (0.25% (v/v) Triton X-100 in PBS), and twice with PBS, sections were stained with corresponding Alexa Fluor 488- or Alexa Fluor 594-conjugated secondary antibodies (Thermo Fisher Scientific) in 1% (v/v)

normal goat serum, 1% (v/v) BSA, and PBS for 1 h at room temperature. After another wash with washing buffer and PBS, the slides were incubated with the nuclear dye DAPI (4′,6-diamidino-2-phenylindole) in PBS for 5 min. After a final wash with ddH_2O, the slides were coverslipped with Fluoromount medium (Sigma) and visualized with an LSM 710 confocal laser-scanning microscope (Carl Zeiss). To quantify plasma staining, we stained five brainstem sections from both a mouse with pathology and a healthy animal, using the primary antibody of a fully vaccinated mouse at a 1:100 dilution. We visualized the staining using an LSM 710 confocal laser-scanning microscope (Carl Zeiss) at a 40x magnification. We took 10 images of defined areas from each section. The stained cells were counted using ImageJ, and the number of positive cells per square millimeter was calculated.

Atomic force microscopy

Atomic force microscopy was used to evaluate the length distribution of sonicated α-syn fibrils. A volume of 5 μL of sonicated fibrils was applied to a mica slide and incubated for 15 min. The slide was washed three times with 100 μL H_2O and dried with N_2. The sample was measured using a NanoWizard III (JPK BioAFM) with an OMCL-AC160TS cantilever (Olympus) in the tapping mode in air. To determine the length distribution, the fibrils were analyzed using ImageJ. The length of each fibril was measured using the ruler tool.

Transmission electron microscopy of HET-s-derived fibrils

To confirm fibrilization of the four vaccine candidates, negative staining transmission electron microscopy was used to visualize and image the purified samples. Carbon-coated copper grids with a mesh size of 200 squares were discharged at 15 mA, 0.39 mBar for 1 min. An aliquot of 5 μL of ~1 mg/mL was added and allowed to absorb on the grid for 1 min and washed with two drops (50 μL) of ammonium acetate before staining with two drops (50 μL) of 2% filtered uranyl acetate (Electron Microscopy Sciences, Hatfield, PA). The grids were then blotted dry with filter paper, stored at RT, and visualized using a bottom-mounted Eagle 4k x 4k camera on a Tecnai F20 TEM (FEI Company, Hillsboro, OR) operating at 200 kV.

RT-QuIC assay

The K23Q mutant of α-syn was monomerized at 4 °C and 14,000 rpm for 15 min using Amicon Ultra 0.5 mL centrifugal filter devices with a 30 kDa cutoff (Merck). Wells in a 96-well clear bottom plate (Thermo Fisher Scientific) were preloaded with six 0.8 mm diameter silica beads (OPS Diagnostic) and filled with 98 µL of reaction buffer containing 40 mM phosphate buffer, 170 mM sodium chloride, 10 µM thioflavin T, and 1 mg/mL mutant α-syn. Positive control seeding wells were incubated with 2 µL of synthetic human wild-type α-syn fibrils (8 nM monomer equivalent). Test wells were incubated with 2 µL of HET-s-derived fibrils (8 nM monomer equivalent). Negative control wells were incubated with 2 µL phosphate buffered saline. Finally, the microplate was sealed with a protective film and incubated on a Clariostar microplate reader (BMG Labtech) at 42 °C with 1 min cycles of double orbital shaking at 400 rpm and 1 min rest. Thioflavin T fluorescence was measured at 450/480 nm every 45 min for 50 h.

Cell assay for α-syn aggregation

A construct encoding human α-syn with the familial A53T mutation fused to enhanced yellow fluorescent protein (YFP) at the C-terminus was custom-ordered (Thermo Fisher Scientific) and subcloned into the pIRESpuro3 vector (Takara Bio) using the NheI (5′) and NotI (3′) restriction sites. HEK293T cells (American Type Culture Collection) were cultured in high-glucose Dulbecco's modified Eagle's medium (DMEM, Sigma-Aldrich) supplemented with 10 % (v/v) fetal calf serum (Sigma-Aldrich), 50 units/mL penicillin, and 50 µg/mL streptomycin (Sigma-Aldrich) in a humidified atmosphere of 5 % CO_2 at 37 °C. Cells were transfected with the vector using Lipofectamine 2000 (Thermo Fisher Scientific), and stable cells were selected in medium containing 1 µg/mL puromycin (EMD Millipore). Monoclonal cell lines were generated by fluorescence-activated cell sorting of a polyclonal cell population in 96-well plates using a MoFlo XDP cell sorter (Beckman Coulter). Clone B5 was selected from 24 clonal cell lines and is referred to as α-synA53T–YFP cells. The α-synA53T–YFP cells were plated in a 384-well plate coated with poly-D-lysine (Greiner) at a density of 1,000 cells in 70 µL per well with 0.1 µg/ml Hoechst 33342 (Thermo Fisher Scientific). To seed cellular aggregation of α-syn in α-

synA53T–YFP cells, 50 nM (monomer equivalent) of α-syn fibrils or one of the four HET-s-derived vaccine candidates was incubated with 1.5 % Lipofectamine in OptiMEM for 2 h at room temperature, and 10 µL of this mixture was added to each well 4 h after plating. Four wells were imaged for each condition by taking 16 images per well with an IN Cell Analyzer 6500HS System (Cytiva, SE) using the blue and green fluorescence channels. Image analysis was performed using IN Carta image analysis software (Cytiva, SE) after establishing an automated algorithm to identify intracellular aggregates in live cells.

Immunohistochemical analysis

For immunohistochemical analysis, formalin-fixed tissues were dehydrated in a series of graded alcohol baths and finally in ROTI-Histol (Carl Roth). Tissues were embedded in paraffin, cut into 8-µm-thick coronal sections, mounted on slides, dried overnight, and stored at 4 °C. Tissue sections were first deparaffinized and then rehydrated. For antigen retrieval, slides were incubated in citrate buffer for 10 min and then boiled in a microwave oven for 10 min. After cooling, endogenous peroxidases were deactivated by incubation in 5% hydrogen peroxide in methanol for 30 min. Sections were blocked in 20% (v/v) normal goat serum, 1% (v/v) BSA, and 0.5% Triton X-100 (Sigma) in PBS for 1 h at room temperature. α-Syn phosphorylated at serine 129 was detected by exposure to a 1:1000 dilution of biotinylated pSyn#64 antibody (Wako) in 1% (v/v) normal goat serum, 1% (v/v) BSA, and 0.25% Triton X-100 in PBS overnight at room temperature. For sections stained with plasma, a 1:100 dilution of plasma was used followed by a 1:200 dilution of biotinylated secondary anti-mouse antibody (Vector Laboratories). The sections were washed once with wash buffer (0.25% (v/v) Triton X-100 in PBS) and twice with PBS, and incubated with a mixture of reagents A and B from the MOM kit (Vector Laboratories) for 1 h at room temperature. After another wash, peroxidase activity was visualized with DAB (3-3′-diaminobenzidine) for 40 s. The reaction was stopped with 3% hydrogen peroxide and washed three times with ddH_2O. To visualize nuclei, sections were counterstained with Meyer's hematoxylin (Carl Roth) for 6 min. After a final wash under running tap water for 15 min, sections were coverslipped with Aqua-Poly/Mount (Polysciences) and imaged a Leica DM 6000 B microscope and Leica Application Suite 4.0 (Leica). Paraffin blocks of human brain samples were obtained from The Netherlands Brain Bank, Netherlands Institute for Neuroscience,

Amsterdam (open access www.brainbank.nl). All Material has been collected from donors for or from whom a written informed consent for a brain autopsy and the use of the material and clinical information for research purposes had been obtained by the NBB.

Western blot analysis

Brain and spinal cord samples were homogenized in Ca^{2+} and Mg^{2+}-free PBS (pH 7.4) containing HALT protease and phosphatase inhibitor cocktail (Thermo Fisher Scientific) and benzonase (Thermo Fisher Scientific) using two 30-s cycles in a Precellys 24-Dual homogenizer (Peqlab) to a final concentration of 20% (w/v). Debris was removed by centrifugation at 1000 × g for 5 min at 4 °C. Total protein concentration was determined using the Pierce BCA Protein Assay Kit (Thermo Fisher Scientific). For further analysis, brain homogenates containing 1 mg total protein were adjusted to 750 mM NaCl and incubated with 10% (w/v) N-lauroylsarcosyl (Sigma) for 15 min on ice. Homogenates were ultracentrifuged at 465,000 × g for 1 h at 4 °C over a 3 mL cushion of 10% (w/v) sucrose in a TLA-110 rotor (Beckman Coulter). The resulting pellets were resuspended in 50 µL of fresh TD4215 denaturing buffer containing 4% SDS, 2% β-mercaptoethanol, 192 mM glycine, 25 mM Tris, 5% (w/v) sucrose, HALT protease and phosphatase inhibitor cocktail and benzonase. Samples were boiled for 5 min and loaded onto 4–12% NuPage Bis–Tris gels (Thermo Fisher Scientific) for SDS-polyacrylamide gel electrophoresis in a 2-(N-morpholine)ethanesulfonic acid buffer system (Thermo Fisher Scientific). Proteins were separated and transferred to polyvinylidene difluoride membranes using a semidry blotting system, then cross-linked with 0.4% (v/v) paraformaldehyde in Tris-buffered saline (Sigma) for 30 min at room temperature. The membranes were blocked in a TBS buffer containing 0.05% (v/v) Tween 20 (MP Biomedical) and 5% (w/v) milk for 1 h at room temperature and then incubated overnight at 4 °C with EP1536Y antibody for phosphorylated α-syn (Abcam). After three washes with 0.05% (v/v) Tween 20 in TBS, the blots were incubated with a 1:10,000 dilution of an anti-rabbit horseradish peroxidase-linked secondary antibody (Cayman) for 1 h at room temperature. The chemiluminescence signal was visualized with SuperSignal West Dura Extended Duration Substrate (Thermo Fisher Scientific) in a chemiluminescence reader (Gel Doc XR+ Imaging System, Bio-Rad).

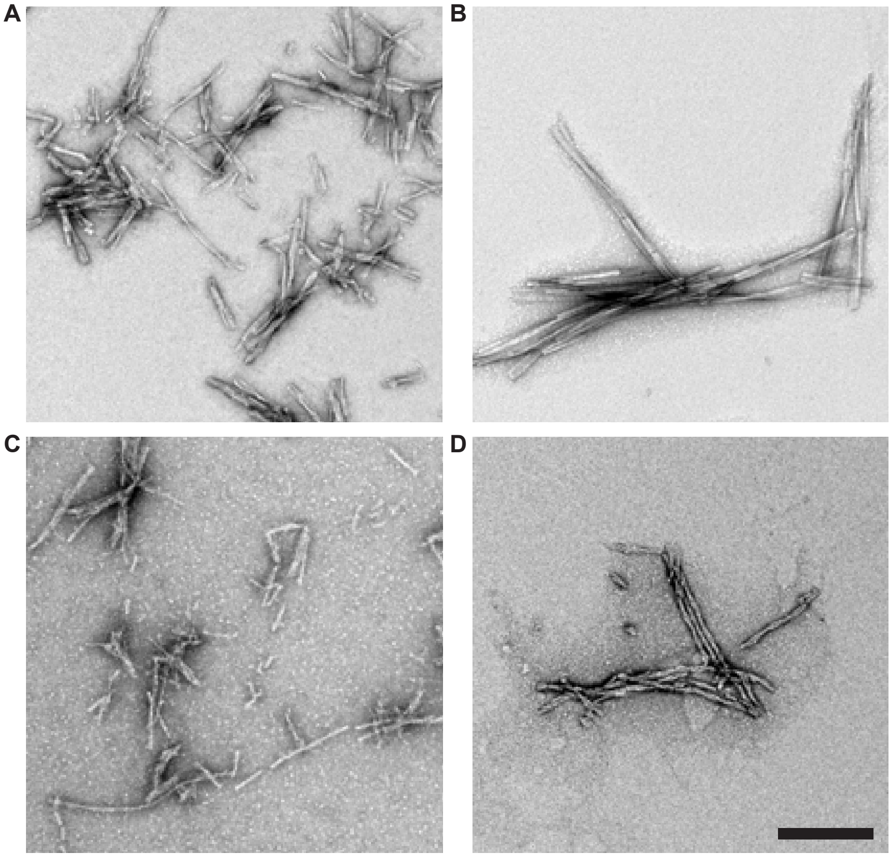

Supplementary Figure 1 HET-s-derived vaccine candidates form fibrils. (**A** to **D**) Negative stain transmission electron microscopy revealed that all four vaccine candidates, **A** α-SC3, **B** α-SC6, **C** α-SC8, and **D** α-SC9, readily formed fibrils. The scale bar represents 200 nm and applies to all panels.

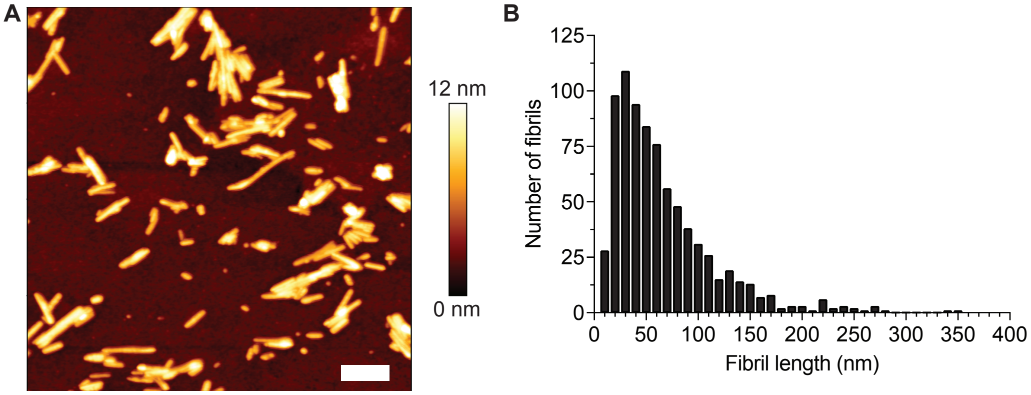

Supplementary Figure 2 Atomic force microscopic analysis of fragment lengths of sonicated α-syn fibrils. (**A**) Sonicated N-acetylated human wild-type α-syn fibrils were imaged by atomic force microscopy. The color scale indicates the height profile. The scale bar represents 250 nm. (**B**) The size distribution of sonicated α-syn fibrils was quantified by measuring the size of each fibril using ImageJ software.

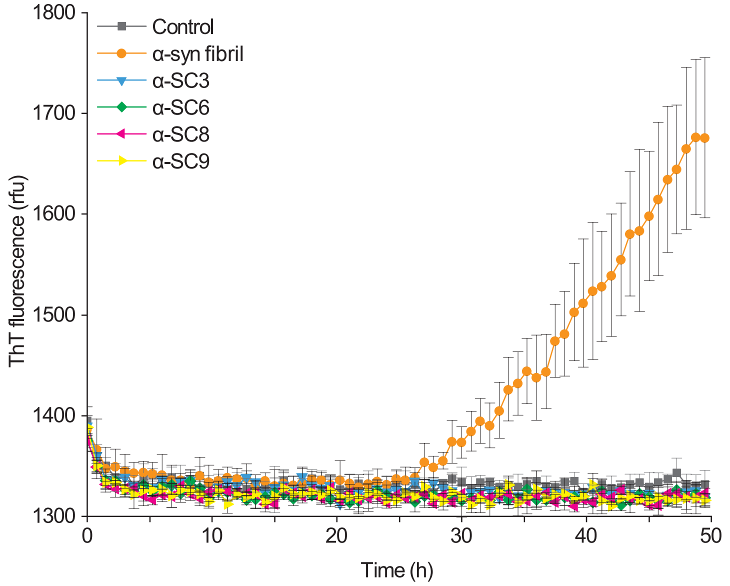

Supplementary Figure 3 The vaccine candidates do not seed aggregation of monomeric α-syn in an RT-QuIC assay. Aggregation of monomeric α-syn was seeded with 160 pM (final monomer equivalent) of sonicated N-acetylated human wild-type α-syn fibrils (orange circles) and resulted in a steady increase in thioflavin T fluorescence beginning at approximately 25 h. In contrast, 160 pM (final monomer equivalent) of sonicated α-SC3 fibrils (blue triangles), α-SC6 fibrils (green diamonds), α-SC8 fibrils (magenta triangles), or α-SC9 fibrils (yellow triangles) did not seed aggregation of monomeric α-syn within 50 h. Aggregation of α-syn monomers was also not observed without seeding (black squares).

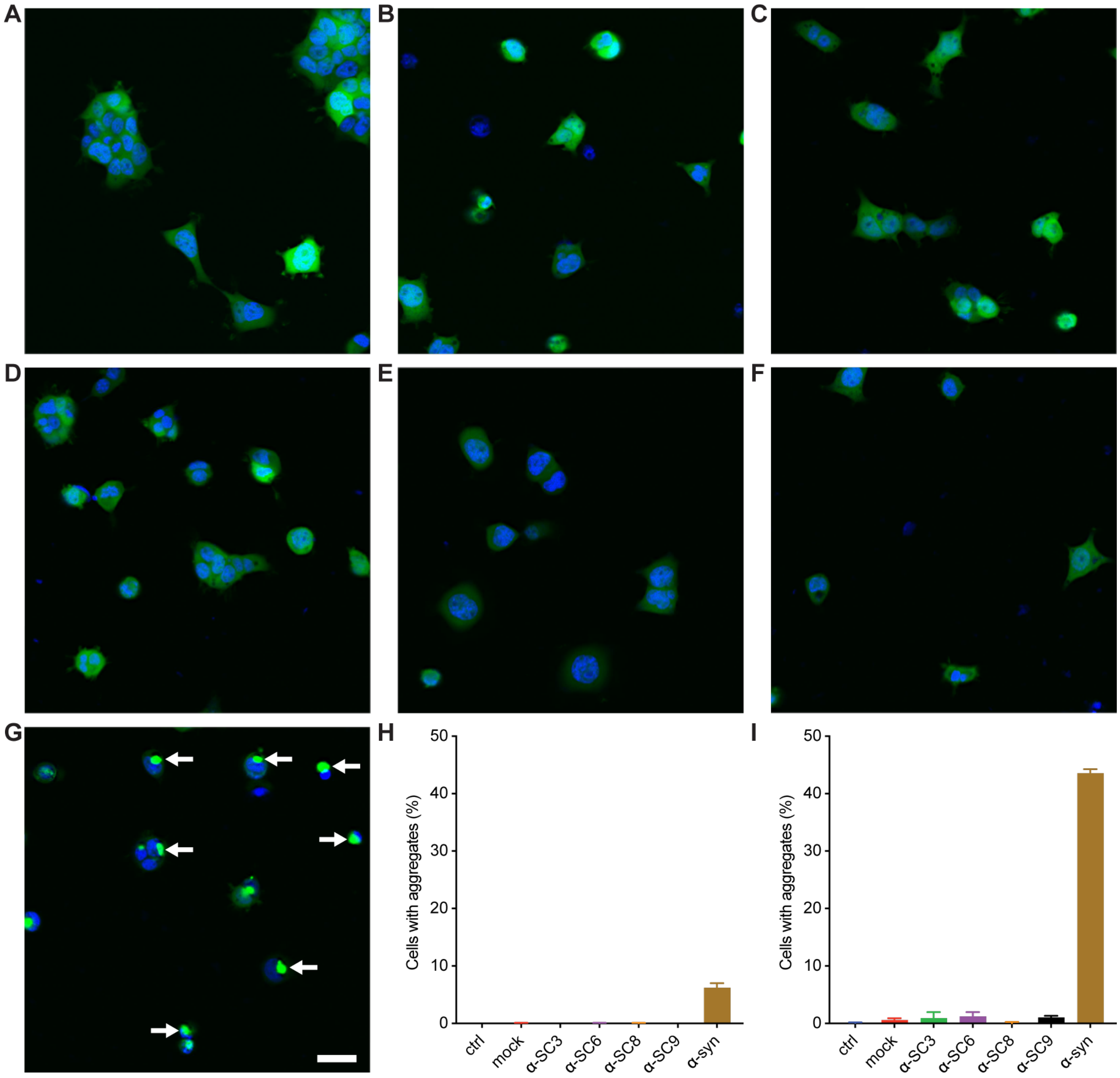

Supplementary Figure 4 HET-s-derived vaccine candidates do not seed aggregation of α-syn in a cell assay. The ability of the four HET-s-derived vaccine candidates to seed aggregation of α-syn was assessed in HEK293T cells expressing α-synA53T–YFP.[2] (**A** to **G**) Images taken 48 h after seeding are shown. In contrast to **A** non-transfected cells, **B** mock-transfected cells, cells transfected with 50 nM (monomer equivalent) of sonicated **C** α-SC3, **D** α-SC6, **E** α-SC8, or **F** α-SC9, only transfection with 50 nM (monomer equivalent) of sonicated N-acetylated human wild-type α-syn fibrils seeded aggregation of α-synA53T–YFP in cells **G** indicated by white arrows. YFP is shown in green and nuclei in blue. The scale bar represents 20 μm and applies to

panels **A** to **G**. Automated image quantification using an established algorithm showed that only synthetic human α-syn fibrils seeded aggregation α-synA53T–YFP ($P < 0.0001$), which was already visible **H** at 24 h and was **I** further increased at 48 h after seeding. Statistical significance was determined by one-way analysis of variance (ANOVA), followed by post hoc Tukey test. Data are shown as mean ± SD, determined from 16 images per well and four wells for each condition.

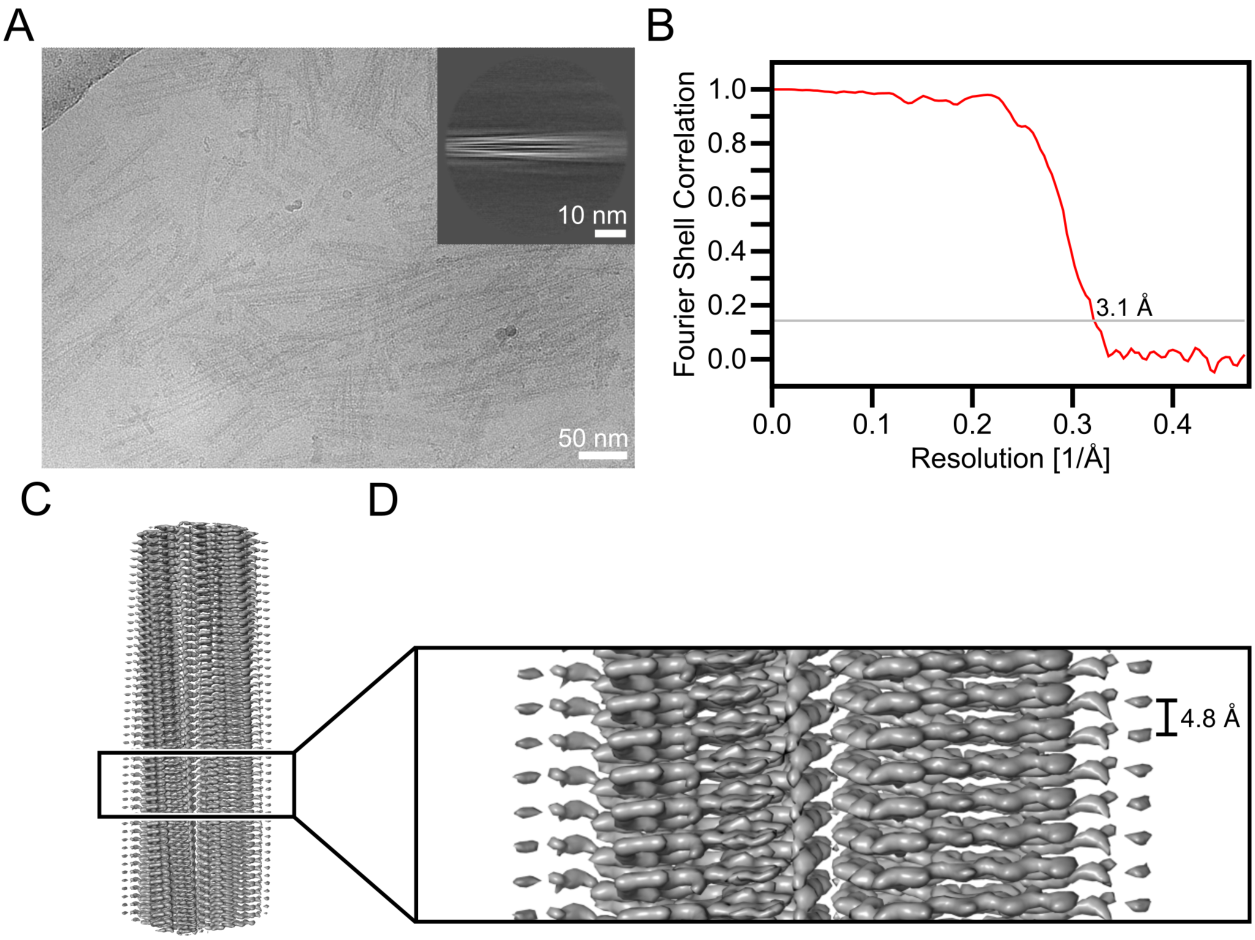

Supplemental Figure 5 Cryo-electron microscopy of N-acetylated human wild-type α-syn fibrils (PDB ID 8OQ1) used to seed α-syn aggregation. (**A**) Representative micrograph. The inset shows a representative 2D class average. (**B**) Masked-corrected (z-percentage is 0.1) Fourier shell correlation (FSC) curve. The final resolution is shown in the plot and was estimated from the value of the FSC curve for two separately refined masked half-maps at 0.143 (gray line). (**C**) Side-view onto the final density map. (**D**) Close-up view onto the final density map showing the staggered arrangement of the protofilaments and the 4.8 Å separation of the stacked β-strands.

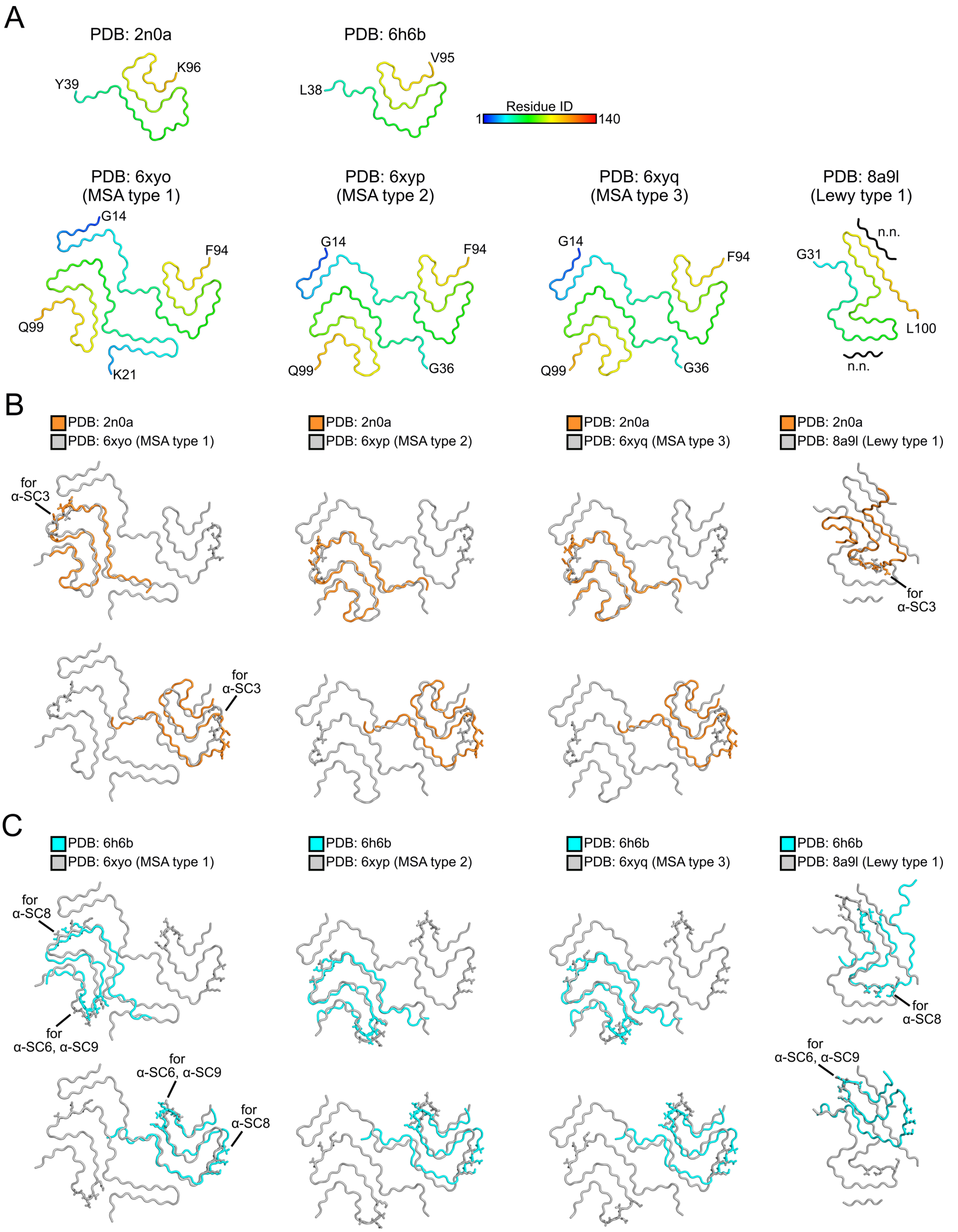
A
PDB: 2n0a
Y39
K96
PDB: 6h6b
L38
V95
Residue ID
1
140
PDB: 6xyo
(MSA type 1)
G14
F94
Q99
K21
PDB: 6xyp
(MSA type 2)
G14
F94
Q99
G36
PDB: 6xyq
(MSA type 3)
G14
F94
Q99
G36
PDB: 8a9l
(Lewy type 1)
n.n.
G31
L100
n.n.
B
PDB: 2n0a
PDB: 6xyo (MSA type 1)
PDB: 2n0a
PDB: 6xyp (MSA type 2)
PDB: 2n0a
PDB: 6xyq (MSA type 3)
PDB: 2n0a
PDB: 8a9l (Lewy type 1)
for
α-SC3
for
α-SC3
for
α-SC3
C
PDB: 6h6b
PDB: 6xyo (MSA type 1)
PDB: 6h6b
PDB: 6xyp (MSA type 2)
PDB: 6h6b
PDB: 6xyq (MSA type 3)
PDB: 6h6b
PDB: 8a9l (Lewy type 1)
for
α-SC8
for
α-SC6, α-SC9
for
α-SC8
for
α-SC6, α-SC9
for
α-SC6, α-SC9
for
α-SC8

Supplementary Figure 6 Comparison of the folds of synthetic and ex vivo α-syn fibrils. (**A**) Shown are ribbons of the protein backbones of the two synthetic α-syn fibrils (PDB IDs 6H6B and 2N0A) that were used to design the four vaccine candidates, and four ex vivo structures, MSA type I to III relevant to MSA, and the Lewy type I fold relevant to PD and DLB. (**B**) Superposition of the protein backbone of the synthetic α-syn fibril with the PDB ID 2N0A (orange) with those of all subunits of the ex vivo fibrils (gray) depicting the residues used to model α-SC3 as sticks. (**C**) Superposition of the protein backbone of the synthetic α-syn fibril with the PDB ID 6H6B (cyan) with those of all subunits of the ex vivo fibrils (gray) depicting the residues used to model α-SC6, α-SC8, and α-SC9 as sticks.

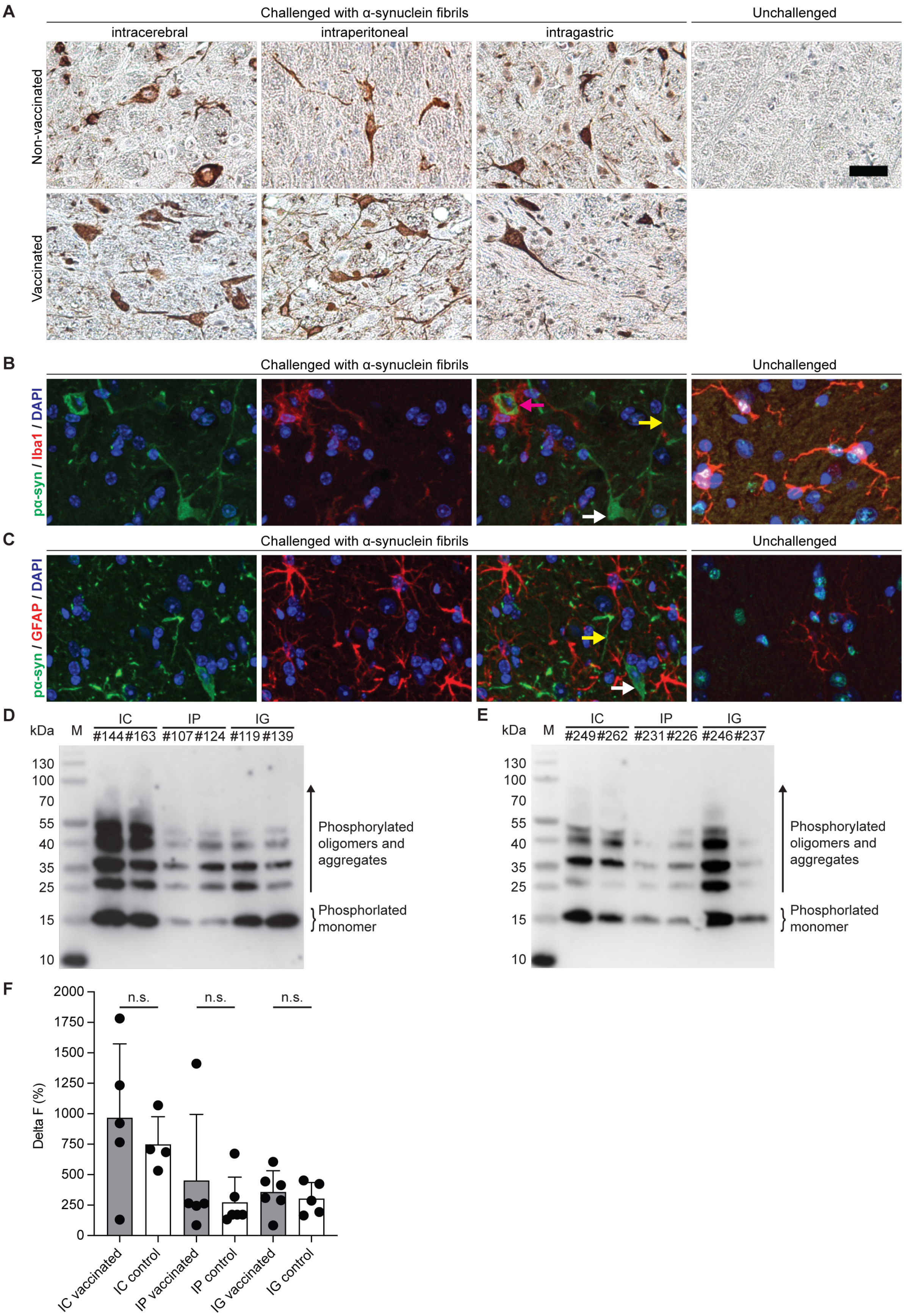
A
Challenged with α-synuclein fibrils
Unchallenged
intracerebral
intraperitoneal
intragastric
Non-vaccinated
Vaccinated
B
Challenged with α-synuclein fibrils
Unchallenged
pα-syn / Iba1 / DAPI
C
Challenged with α-synuclein fibrils
Unchallenged
pα-syn / GFAP / DAPI
D
kDa
M
IC
IP
IG
#144
#163
#107
#124
#119
#139
130
100
70
55
40
35
25
15
10
Phosphorylated oligomers and aggregates
Phosphorlated monomer
E
kDa
M
IC
IP
IG
#249
#262
#231
#226
#246
#237
130
100
70
55
40
35
25
15
10
Phosphorylated oligomers and aggregates
Phosphorlated monomer
F
Delta F (%)
2000
1750
1500
1250
1000
750
500
250
0
n.s.
n.s.
n.s.
IC vaccinated
IC control
IP vaccinated
IP control
IG vaccinated
IG control

Supplementary Figure 7 Vaccination delays but does not prevent neuropathology in mice challenged with α-syn fibrils. (**A**) Immunohistochemical analysis of brainstem sections from TgM83$^{+/-}$ mice with an antibody against α-syn phosphorylated at serine 129 revealed neuronal and neuritic deposits of pathological α-syn aggregates regardless of vaccination status or route of challenge with α-syn fibrils. Mice not challenged with α-syn fibrils showed no neuropathology. The scale bar represents 50 µm and applies to all panels. (**B**) Immunofluorescence analysis of brainstem sections from diseased TgM83$^{+/-}$ mice with antibodies against ionized calcium-binding adaptor molecule 1 (Iba1, red) and α-syn phosphorylated at serine 129 (green) revealed neuronal (white arrow) and neuritic (yellow arrow) deposits surrounded (magenta arrow) by activated, amoeboid microglia, regardless of vaccination status or route of challenge with α-syn fibrils. Unchallenged age-matched mice showed no pathology and predominantly quiescent, ramified microglia. Nuclei were stained with DAPI in blue. (**C**) Immunofluorescence analysis of midbrain tissue sections of diseased TgM83$^{+/-}$ mice with antibodies against glial fibrillary acidic protein (GFAP, red) and α-syn phosphorylated at serine 129 (green) revealed neuronal (white arrow) and neuritic (yellow arrow) deposits and an accumulation of astrocytes indicative of astrogliosis regardless of the vaccination status or route of challenge with α-syn fibrils. (**D** and **E**) Unchallenged mice did not reveal any pathology. Nuclei were stained blue with DAPI. Brain homogenates from diseased TgM83$^{+/-}$ mice had accumulated high molecular weight species of α-syn phosphorylated at serine 129 (**D**) regardless of whether they were vaccinated, (**E**) unvaccinated, or the route of challenge with α-syn fibrils. (**F**) The amount of α-syn aggregates in brain homogenates of terminally sick animals did not significantly differ based on their vaccination status. Bars represent mean ± SD. *ns* = not significant.

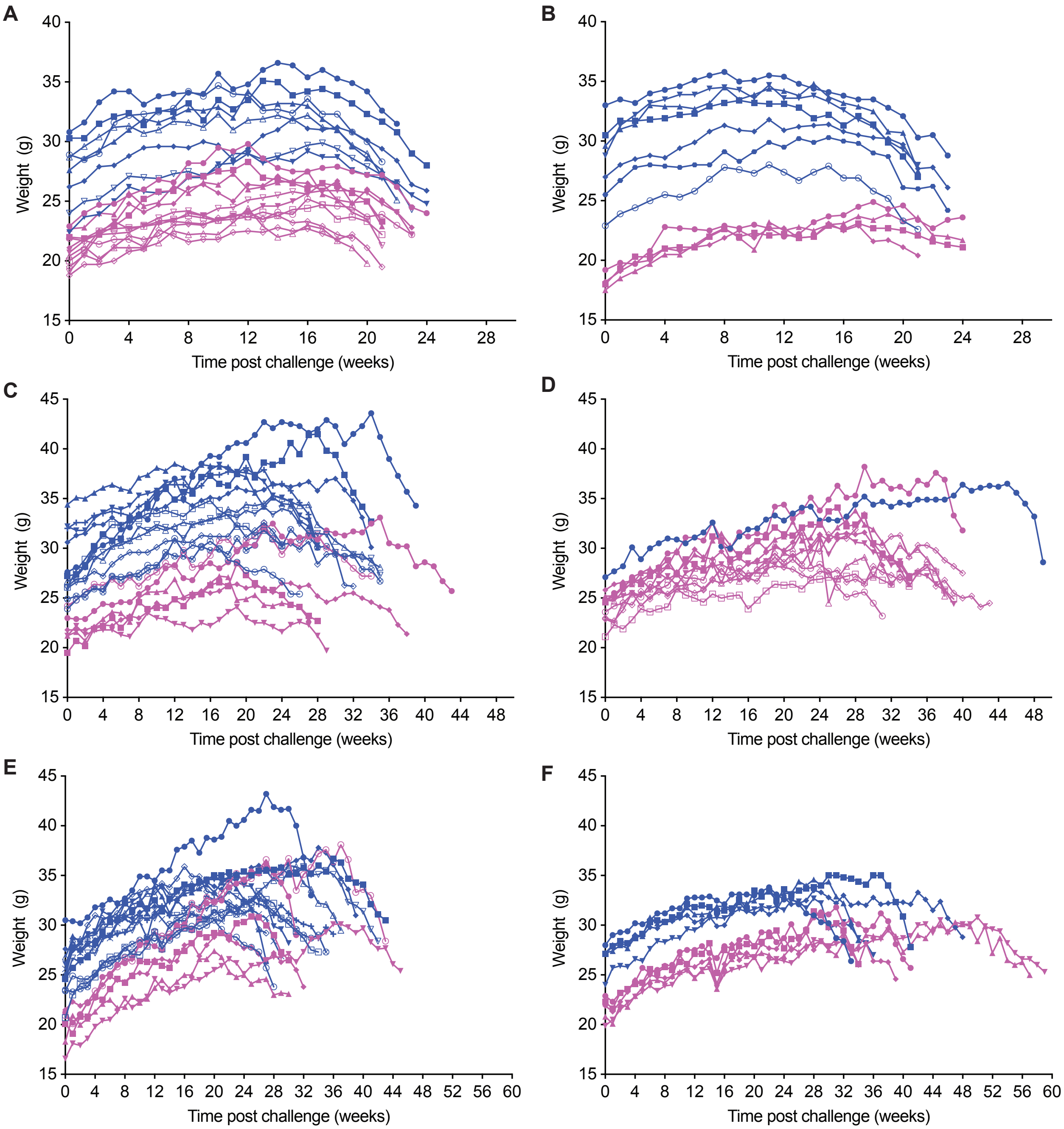

Supplementary Figure 8 Weights of TgM83$^{+/-}$ mice injected with α-syn fibrils. (**A** and **B**) Shown are the weights of **A** immunized and **B** non-immunized control mice after intracerebral injection of α-syn fibrils. (**C** and **D**) Shown are the weights of **C** immunized and **D** non-immunized control mice after intraperitoneal injection of α-syn fibrils. (**E** and **F**) Shown are the weights of **E** immunized and **F** non-immunized control mice after injection of α-syn fibrils into

the intestinal wall. The weight of male mice is shown in blue and the weight of female mice is shown in magenta.

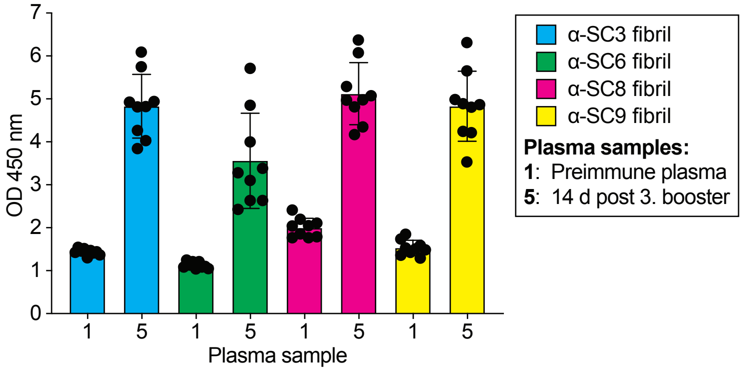

Supplementary Figure 9 Immunization with the vaccine candidates induces antibodies that recognize all four vaccine candidates. ELISA measurements of plasma collected from nine TgM83$^{+/-}$ mice showed that in contrast to preimmune plasma (1:10,000 dilution) collected from unvaccinated mice, plasma (1:330,000 dilution) collected from fully immunized mice two weeks after the third booster dose had significantly higher antibody titers to each of the four vaccine candidates. Bars represent mean ± SD.

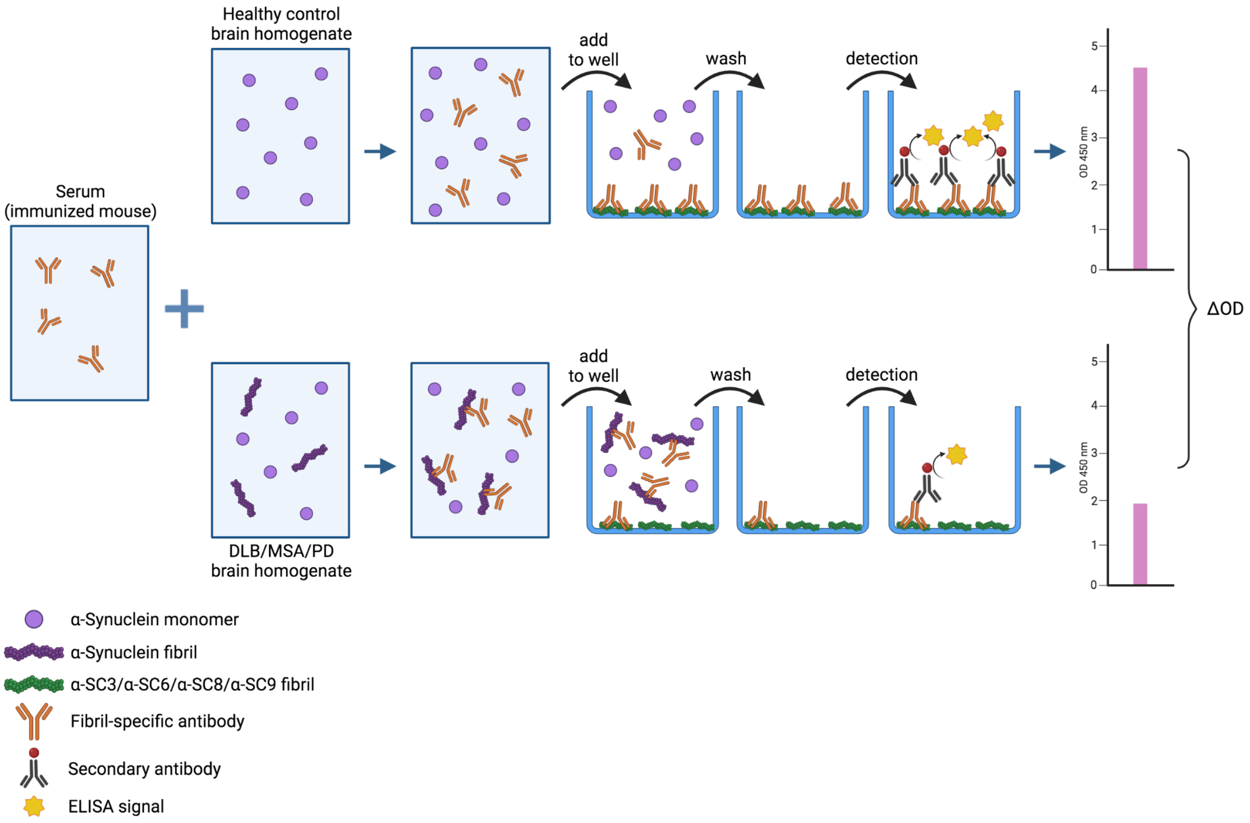

Supplementary Figure 10 Principle of the competitive ELISA. For the competitive ELISA, plasma from immunized mice was mixed with brain homogenates from healthy controls and patients with DLB, MSA, or PD. Since brain homogenates from healthy controls contain only monomeric α-syn and no α-syn fibrils, most of the antibodies in the plasma of immunized mice are free to bind to wells precoated with α-SC3, α-SC6, α-SC8, or α-SC9 fibrils, resulting in a relatively high ELISA signal [OD 450 nm(HC)]. In contrast, brain homogenates from patients with synucleinopathies contain pathological α-syn fibrils that are bound by antibodies in the plasma of immunized mice, thereby reducing the number of antibodies that are free to bind to wells precoated with α-SC3, α-SC6, α-SC8, or α-SC9 fibrils, resulting in a relatively low ELISA signal [OD 450 nm(DLB/MSA/PD)]. The competitive ELISA signal ΔOD 450 nm, is the difference between the two ELISA signals [OD 450 nm(HC) − OD 450 nm(DLB/MSA/PD)]. ΔOD 450 nm values greater than zero indicate the presence of antibodies in the plasma of fully immunized mice that recognize pathological α-syn fibrils.

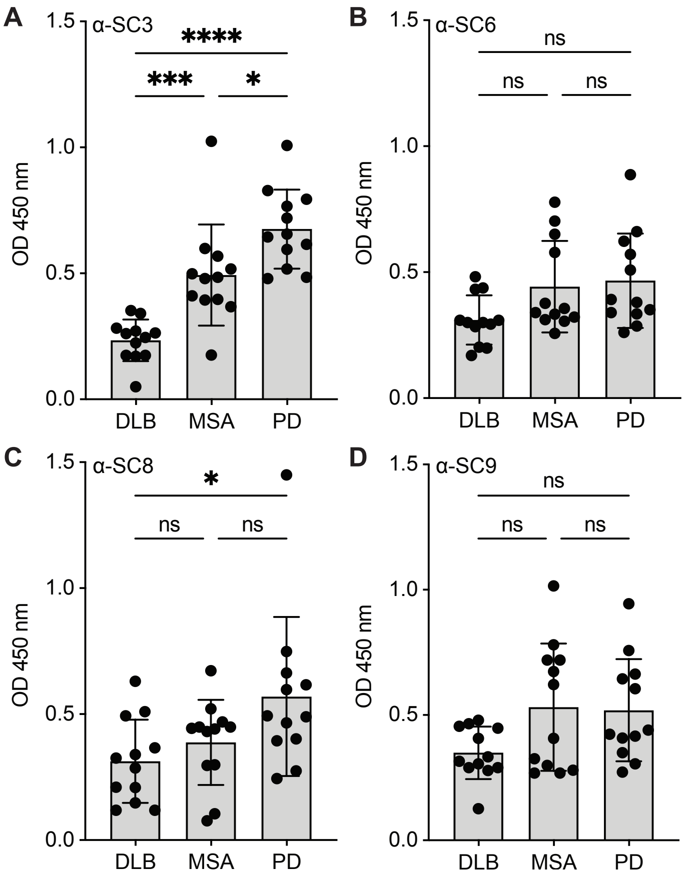

Supplementary Figure 11 The four vaccine candidates induce antibodies with varying affinities towards different human synucleinopathies as detected by TR-FRET. All four vaccine candidates induced antibodies that recognized patient brain homogenates of all three synucleinopathies better than the healthy control brain homogenates. However, the four vaccine candidates also showed different efficacies against different disease-derived fibrils. For example, α-SC3 (**A**) and α-SC8 (**C**) induced a significantly ($p < 0.05$) stronger antibody response for PD than for multiple system atrophy or dementia with Lewy bodies compared to α-SC6 (**B**) and α-SC9 (**D**) (Supplementary Fig. 11). Bars represent mean ± SD. *ns* = not significant, *$P < 0.05$, ***$P < 0.001$, ****$P < 0.0001$.

Supplementary Table 1 Cryo-EM structure determination statistics

**Data Collection**	
Microscope	Titan Krios G4
Voltage [keV]	300
Detector	K3
Magnification	81,000
Pixel size [Å]	1.06
Defocus range [µm]	−0.5 to −2.5
Exposure time [s]	1.2
Number of frames	30
Total dose [e^-/Å^2]	~30.5
**Reconstruction**	
Micrographs	7,316
Box width [pixels]	250
Inter-box distance [pixels]	13
Picked segments (no.)	1,590,869
**Final map**	
EMDB-ID	EMD-17111
Final segments [no.]	27,610
Final resolution [Å] (FSC = 0.143)	3.1
Applied map sharpening B-factor [Å^2]	−105
Symmetry imposed	C1
Helical rise [Å]	2.41
Helical twist [°]	179.62

Supplementary Table 2 Model building statistics

**Initial model [PDB code]**	6H6B[1]
**Model composition**	
PDB-ID	8OQI
Chains	10
Non-hydrogen atoms	3,810
Protein residues	560
**RMS deviations**	
Bond lengths [Å]	0.01
Bond angles [°]	2.01
**Validation**	
MolProbity score	1.75
Clashscore	7.89
**Ramachandran plot**	
Outliers [%]	0.00
Allowed [%]	0.00
Favored [%]	100.00

Supplementary Table 3 Human brain tissues used for immunohistochemistry

Sample ID	Diagnosis	Sex	Age	Brain region	Braak LB stage
PDD	PDD	m	72	Substantia nigra	6
DLB	DLB	m	58	Substantia nigra	6

PDD Parkinson's disease with dementia, *DLB* dementia with Lewy bodies, *m* male

Supplementary Table 4 Human brain tissues used for competitive ELISA

Sample ID	Diagnosis	Sex	Age	Brain region	Braak LB stage
HC1	HC	m	72	Medulla oblongata	0
HC2	HC	f	80	Medulla oblongata	0
HC3	HC	f	55	Substantia nigra	0
HC4	HC	f	78	Putamen	0
HC5	HC	m	69	Medulla oblongata	0
DLB1	DLB	f	86	Caudate with putamen	5
DLB2	DLB	m	78	Caudate with putamen accumbens	6
DLB3	DLB	m	72	Caudate with putamen accumbens	6
MSA1	MSA	f	61	Cerebellum	–
MSA2	MSA	m	57	Cerebellum	–
MSA3	MSA	m	73	Cerebellum	–
PD1	PD	m	72	Caudate with putamen accumbens	5
PD2	PD	m	76	Caudate with putamen accumbens	6
PD3	PD	m	82	Caudate with putamen accumbens	6

HC healthy control, *DLB* dementia with Lewy bodies, *MSA* multiple system atrophy, *PD* Parkinson's disease, *m* male, *f* female, *LB* Lewy body

References

1. Guerrero-Ferreira R, Taylor NMI, Mona D*, et al*. Cryo-EM structure of alpha-synuclein fibrils. *Elife*. Jul 3 2018;7doi:10.7554/eLife.36402
2. Woerman AL, Stohr J, Aoyagi A*, et al*. Propagation of prions causing synucleinopathies in cultured cells. *Proc Natl Acad Sci U S A*. Sep 1 2015;112(35):E4949-E4958. doi:10.1073/pnas.1513426112
